# Training-Free Skeleton-Semantic Keyframe Extraction for Fixed-View Industrial Assembly Video: Method Design and Case Study Evaluation

**DOI:** 10.3390/s26154956

**Published:** 2026-08-05

**Authors:** Qianhui Li, Hua Xiang, Tongxi Wang, Wei Zhan

**Affiliations:** 1 School of Computer Science, Yangtze University, Jingzhou 434023, China; 2024720787@yangtzeu.edu.cn (Q.L.); 300546@yangtzeu.edu.cn (T.W.); zhanwei814@yangtzeu.edu.cn (W.Z.); 2 Research Center for Digital Agriculture and Intelligent Engineering, Yangtze University, Jingzhou 434023, China; 3 Artificial Intelligence Research Platform, Yangtze University, Jingzhou 434023, China

**Keywords:** industrial assembly video, keyframe extraction, human pose semantics, skeleton semantic features, adaptive threshold

## Abstract

**Highlights:**

**What are the main findings?**

- A deterministic, training-free framework converts a continuous fixed-view assembly video into a controllable sequence of pose-informative keyframes.
- At matched budgets, the selected frames show substantially more regular skeleton-semantic increments and greater pose-state separation than the evaluated temporal, pixel, clustering, and pretrained MViT baselines.

**What are the implications of the main findings?**

- The framework offers an interpretable preprocessing option when the objective is compact coverage of distinct operator-pose states without task-specific model training.
- Ablation and missing-data analyses identify the skeleton representation as the main contributor and define how adaptive thresholding and interpolation affect the measured behavior.

**Abstract:**

Industrial assembly videos contain substantial temporal redundancy, creating a need for preprocessing methods that remain usable when task-specific labels, retraining, or opaque learned selection rules are undesirable. This study designs a deterministic, training-free skeleton-semantic keyframe extraction framework for fixed-view monocular assembly video preprocessing. OpenPose BODY-25 keypoints are confidence-filtered, normalized, interpolated, and represented by eight upper-body joints. Neck-relative bilateral-wrist activity controls an inverse threshold, while sequential reference updates and a maximum-gap rule provide an explicit output budget. At 271 frames per analyzed file, the method obtains CVsd = 0.3340 ± 0.1117 and MSD = 0.7781 ± 0.1003, compared with 1.0681 ± 0.0754 and 0.2961 ± 0.0305 for pretrained MViT-V2-S feature clustering. Thus, under the shared normalized skeleton evaluation, the method reduces semantic-distance variation by 68.7% and increases adjacent pose-state separation by 162.8% relative to MViT. Matched ablation identifies the skeleton representation as the primary source of this organization; adaptive thresholding provides smaller, budget-dependent density adjustment. A second independent human annotation and adjudication establish a 34-transition event reference. Event results reveal a complementary trade-off: the proposed method retains 79.41% of transitions within ±0.5 s, whereas several temporal and appearance-based baselines retain 97.06–100%, showing that pose-state diversity and process-boundary coverage are different sampling objectives. Matched-baseline observed-joint-only and confidence-weighted analyses show that interpolation contributes to numerical regularity, but the proposed method retains substantially lower semantic-distance variation and greater adjacent pose-state separation than all evaluated baselines under both missing-data treatments. These results establish the method as an interpretable, budget-controllable preprocessing strategy for pose-oriented review and data reduction in the evaluated fixed-view setting. Validation across independently recorded production environments and task-specific downstream systems is the next step toward broader deployment.

## 1. Introduction

Video monitoring systems are widely used in industrial assembly lines for process supervision, quality inspection, production traceability, and downstream intelligent manufacturing tasks. Recent industrial and assembly-oriented studies have investigated action recognition, progress prediction, multi-view procedural understanding, egocentric human–object interaction, and pose-aware assembly analysis [1,2,3,4,5]. Continuous camera-based monitoring produces long video streams, and processing every frame increases transmission, storage, and computational costs, particularly in edge-computing environments [6]. Video summarization and adaptive frame-selection research therefore aims to reduce temporal redundancy while retaining representative information for subsequent analysis [7,8,9,10].

Existing keyframe extraction and video summarization methods commonly rely on pixel changes, local descriptors, optical flow, learned importance scores, or pretrained video features. Complementary video-analysis research has combined spatial, frequency-domain, and optical-flow cues for AIGC video detection [11] and developed adaptive sparse memory for efficient and robust video object segmentation [12]. Although these studies address tasks different from keyframe extraction, they illustrate broader strategies for integrating complementary visual-temporal cues and controlling redundant video information. These approaches serve different objectives, but they present two practical gaps for fixed-view industrial monitoring. First, appearance changes caused by reflections, illumination, tools, or background activity need not correspond to a change in the operator’s pose state. Second, learned summarizers may require annotated task data, fine-tuning, or an output objective that is difficult to audit and control. Skeleton representations offer a compact description of human configuration, while a deterministic sampler offers reproducible decisions and a directly specified storage budget. Prior skeleton research has concentrated mainly on recognition models [13,14,15,16,17]; comparatively less attention has been given to using pose semantics as the decision space for transparent, training-free keyframe preprocessing.

The engineering question addressed here is therefore not whether one sampler is universally best, but whether a simple, auditable mechanism can deliberately preserve differentiated operator-pose states under a fixed output budget. This objective motivates potential use in pose-oriented review, representative-frame browsing, annotation preparation, and storage reduction in stable-camera workstations, where retaining another visually similar frame may be less informative than retaining a new arm–wrist configuration. These downstream uses remain hypotheses rather than validated outcomes. The framework also provides a reproducible reference point against which learned or event-aware selectors can later be compared.

To address this question, this paper designs an offline skeleton-semantic adaptive sampler for a practical fixed monocular camera configuration. The framework integrates four conventional but complementary operations: normalized upper-body pose representation, confidence-aware bilateral-wrist activity relative to a body anchor, inverse activity-to-threshold mapping, and sequential selection with a maximum-gap constraint. Its contribution is a reproducible decision pipeline that needs no task-specific training, exposes the sampling criterion, and permits direct control of output capacity. The study then separates three evaluation questions—pose-state organization, trajectory/event coverage, and sensitivity to missing keypoints—so that the method’s intended benefit is not conflated with objectives it was not designed to optimize.

The main contributions of this study are summarized as follows:

- A task-specific, 16-dimensional upper-body representation and sequential reference-update rule are defined to select frames according to operator-pose evolution rather than raw appearance change. The complete procedure is deterministic and requires no task-specific training labels.
- A bilateral-wrist, anchor-relative activity estimate is coupled to an inverse threshold and maximum-gap constraint, providing interpretable temporal density adjustment and explicit control of the keyframe budget.
- Matched-budget comparisons with temporal, pixel, clustering, motion, and pretrained MViT baselines quantify the framework’s distinctive behavior: substantially more regular semantic increments and greater separation between neighboring pose states. Complementary event and reconstruction results clarify when alternative sampling objectives are preferable.
- A two-annotator event reference, matched ablation, parameter sensitivity, anchor analysis, controlled pose degradation, illumination transformations, missing-gap analysis, observed-joint sensitivity, and runtime measurement provide a transparent characterization of both the mechanism and its operating conditions.

The remainder of this paper is organized as follows. Section 2 describes the proposed method, including skeleton feature extraction, relative-motion calculation, adaptive thresholding, and keyframe update rules. Section 3 presents the dataset, evaluation protocol, matched-budget comparisons, ablation and sensitivity analyses, controlled degradation tests, and computational cost. Section 4 discusses the method’s advantages, metric-specific behavior, and current application scope. Section 5 concludes the paper.

## 2. Materials and Methods

### 2.1. Overview of the Proposed Framework

**Figure 1:**
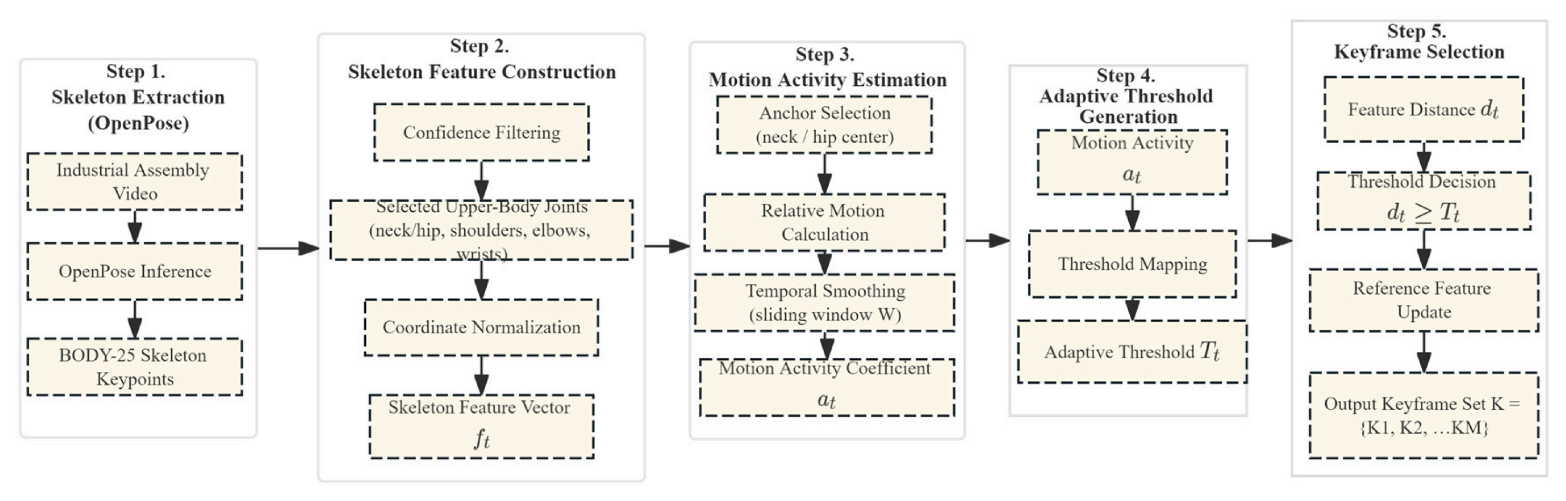
Overall workflow of the proposed skeleton-semantic adaptive keyframe extraction framework for industrial assembly videos. The framework consists of five stages: OpenPose-based BODY-25 skeleton extraction, skeleton feature construction with confidence filtering and coordinate normalization, motion activity estimation using anchor-based relative motion and temporal smoothing, adaptive threshold generation, and keyframe selection based on the comparison between feature distance $d_{t}$ and adaptive threshold $T_{t}$. After a frame is selected, the reference feature is updated for subsequent keyframe decisions. Arrows indicate the processing flow between stages.

As shown in Figure 1, the proposed framework takes a continuous sequence of frames captured by an industrial monitoring camera as input. OpenPose is first used to extract BODY-25 skeleton keypoints from each frame [18], and the extracted keypoints provide the basic representation of the operator’s pose during assembly operations.

The skeleton feature construction stage then performs confidence filtering to remove unreliable keypoints, selects task-related upper-body joints, and applies coordinate normalization to construct the skeleton feature vector $f_{t}$. Based on the normalized skeleton features, an anchor point, either the neck joint or the hip center, is selected for relative motion calculation. The resulting relative motion sequence is smoothed by a sliding window to reduce short-term pose-estimation jitter, and the motion activity coefficient $a_{t}$ is calculated to describe the temporal motion intensity of the assembly operation.The main notation used in the proposed method is summarized in Table 1.

The motion activity coefficient $a_{t}$ is then mapped to an adaptive threshold $T_{t}$, which regulates keyframe selection according to the current motion state. In the keyframe selection stage, the feature distance $d_{t}$ between the current frame and the reference feature is compared with $T_{t}$. If $d_{t}$ satisfies the threshold condition, the current frame is selected as a keyframe and the reference feature is updated. The final output is a set of keyframes K that reduces temporal redundancy while retaining representative skeleton-motion information in the evaluated industrial assembly setting.

### 2.2. OpenPose-Based Skeleton Extraction

#### Posture Feature Extraction

The first stage of the framework extracts the skeleton sequence. Given an industrial assembly video captured by a monocular monitoring camera, the video is first decomposed into a sequence of frames:

(1) $$I=[I_{1},I_{2}\ldots ,I_{N}],$$

where $I_{t}$ denotes the t-th video frame and N denotes the total number of frames.

For each frame, OpenPose is applied to extract the BODY-25 human skeleton representation. The BODY-25 model provides 25 body keypoints for each detected human operator. Each keypoint contains a two-dimensional image coordinate and a confidence score. More recent transformer-based pose estimators demonstrate an alternative direction for generic body-pose estimation [19], while OpenPose is used here to generate the BODY-25 skeleton sequence analyzed in this study. The j-th keypoint in frame t is represented as:

(2) $$P_{t,j}=(x_{t,j},y_{t,j}),c_{t,j}\in [0,\;1],j=0,1,\ldots ,24,$$

where $P_{t,j}$ denotes the two-dimensional coordinate of the j-th BODY-25 keypoint, and $c_{t,j}$ denotes the corresponding confidence score estimated by OpenPose.

In the implementation, the OpenPose output is stored in JSON format for each frame and then converted into a frame-wise skeleton table. Each row of the table corresponds to one video frame and contains the frame index, the x-coordinate, y-coordinate, and confidence score of all 25 BODY-25 keypoints. If no human operator is detected in a frame, the coordinates and confidence scores of the corresponding keypoints are initialized as zero and further handled by the confidence filtering and interpolation procedure in the next stage.

Because the monitored industrial assembly scene contains one primary operator, the first detected person in each frame is used as the target skeleton. This setting is consistent with the single-operator assembly videos used in the experiments. The output of this stage is a temporal skeleton sequence containing BODY-25 keypoints and confidence scores for all video frames, which provides the input for skeleton feature construction.

### 2.3. Skeleton Feature Construction

After obtaining the BODY-25 skeleton sequence from OpenPose, the raw keypoints are processed to construct a compact skeleton feature vector for each frame. The implementation first forms a confidence-validity mask, normalizes the complete coordinate sequence, replaces low-confidence normalized coordinates by temporal interpolation, and then selects the upper-body joints used to construct the feature vector. These operations correspond to Step 2 of Figure 1.

#### 2.3.1. Confidence Filtering and Missing-Keypoint Handling

OpenPose may produce unreliable or missing keypoints under occlusion, tool interference, body rotation, or poor lighting conditions. Occlusion-aware pose-estimation studies explicitly model joint visibility and linked-joint relations to improve reasoning under partial observation [20,21]. In the present preprocessing pipeline, confidence filtering is instead applied to each BODY-25 keypoint. A keypoint is considered valid only when its confidence score is greater than or equal to the predefined confidence threshold:

(3) $$\left\{\begin{array}{cc} v_{t,j}=1, & if\;c_{t,j}\geq c_{min}\\ v_{t,j}=0, & if\;c_{t,j}<c_{min} \end{array}\right.,$$

where the validity indicator specifies whether the j-th keypoint in frame t satisfies the confidence requirement. In this study, $c_{min}$ is set to 0.2.

The confidence mask is retained while the coordinate sequence is normalized as described in Section 2.3.3. After normalization, coordinates whose confidence scores are lower than $c_{min}$ are replaced by temporal linear interpolation from the nearest valid observations of the same joint. If a joint has no valid observation in the entire sequence, its normalized coordinate is set to zero. This fixed processing order is used in all experiments.

#### 2.3.2. Selected Upper-Body Joints

Industrial assembly actions are primarily characterized by upper-body motion, including movements of the shoulders, elbows, wrists, and torso. Therefore, a selected joint index set $J_{f}$ is used for skeleton feature construction:

(4) $$J_{f}=\{1,\;2,\;3,\;4,\;5,\;6,\;7,\;8\},$$

In the BODY-25 format, these indices correspond to the neck, right shoulder, right elbow, right wrist, left shoulder, left elbow, left wrist, and mid-hip joints. This selection retains the principal operator-centered motion information associated with manual assembly while reducing the influence of task-irrelevant lower-body keypoints.

In addition, the wrist-joint set $J_{m}$ is defined for motion activity estimation in the next stage:

(5) $$J_{m}=\{4,7\},$$

where joints 4 and 7 correspond to the right wrist and left wrist, respectively. The two wrist joints are selected because wrist movement is closely related to typical assembly actions such as picking, aligning, placing, and tightening components.

#### 2.3.3. Coordinate Normalization

Before interpolation and selected-joint feature construction, global min–max normalization is applied independently to the $\tilde{x}$ and $\tilde{y}$ coordinate axes over the complete BODY-25 sequence:

(6) $${\tilde{x}}_{t,j}=\frac{x_{t,j}-x_{min}}{x_{max}-x_{min}+10^{-9}},$$

(7) $${\tilde{y}}_{t,j}=\frac{y_{t,j}-y_{min}}{y_{max}-y_{min}+10^{-9}},$$

where $x_{min}$, $x_{max}$, $y_{min}$, and $y_{max}$ are computed from the complete skeleton coordinate sequence. The constant 10^−9^ prevents division by zero. The confidence mask defined in Section 2.3.1 is then used to replace unreliable normalized coordinates by interpolation before the selected joints are concatenated.

#### 2.3.4. Skeleton Feature Vector Construction

Based on the selected joint set $J_{f}$, the skeleton feature vector of frame t is constructed by concatenating the normalized coordinates of all selected joints:

(8) $$f_{t}={\left[{\tilde{x}}_{t,1},{\tilde{y}}_{t,1},{\tilde{x}}_{t,2},{\tilde{y}}_{t,2},\ldots ,{\tilde{x}}_{t,8},{\tilde{y}}_{t,8}\right]}^{⊤},$$

where $f_{t}$ denotes the skeleton feature vector of frame t. Since $J_{f}$ contains eight selected joints and each joint has two coordinate components, $f_{t}$ is a 16-dimensional vector. The resulting feature vector provides a compact representation of operator-centered assembly motion and is used to calculate feature distance during keyframe selection.

### 2.4. Anchor-Based Motion Activity Estimation

This stage estimates the temporal motion intensity of assembly operations from the normalized skeleton sequence. As shown in Step 3 of Figure 1, this stage includes anchor selection, relative motion calculation, temporal smoothing, and motion activity coefficient mapping. Motion activity is calculated from the relative movement of both wrist joints with respect to the selected anchor point.

#### 2.4.1. Anchor Selection

To reduce the influence of body translation and camera disturbance, wrist motion is calculated relative to an anchor point. The neck joint is used as the default anchor in the proposed method because it is generally available in the evaluated upper-body views. Because neck motion during bending or postural changes may distort the relative-motion estimate, the hip center is evaluated as an alternative anchor in a controlled comparison. The default neck anchor is defined as:

(9) $$b_{t}^{neck}=p_{t,1},$$

The hip-center anchor is defined using the mid-hip joint when it is valid. If the mid-hip joint is unavailable, the average of the left and right hip joints is used:

(10) $$b_{t}^{hip}=p_{t,8},\;if\;c_{t,8}\geq c_{min}\;b_{t}^{hip}=\frac{p_{t,9}+p_{t,12}}{2},$$

where the symbols denote the neck, mid-hip, right-hip, and left-hip joints in the BODY-25 format, respectively. Unless an experiment is explicitly labeled as the hip-center variant, all reported proposed-method results use the neck anchor. The anchor comparison quantifies the sensitivity of motion activity estimation to this design choice.

#### 2.4.2. Relative Wrist-Motion Calculation

Wrist movement is strongly associated with manual assembly because actions such as picking, aligning, placing, and tightening are performed primarily by hand. Therefore, the wrist-joint set $J_{m}$ = {4, 7} is used for motion activity estimation, where joints 4 and 7 correspond to the right wrist and left wrist, respectively. For each wrist joint j in $J_{m}$, the anchor-compensated relative displacement between two adjacent frames is calculated as:

(11) $$r_{t,j}=\left(p_{t,j}-p_{t-1,j}\right)-\left(b_{t}-b_{t-1}\right),\;j\in J_{m},$$

The relative motion magnitude of frame t is then computed by averaging the valid wrist motions:

(12) $$m_{t}=mean\left(\|r_{t,j}{\|}_{2}\right),\;j\in J_{m}\;and\;c_{t,j}\geq c_{min},$$

If both wrist joints are invalid in a frame, $m_{t}$ is set to zero. This confidence-aware calculation reduces the influence of wrist-tracking failures while preserving the temporal structure of the skeleton sequence.

#### 2.4.3. Temporal Smoothing and Activity Coefficient Mapping

Because frame-level pose estimation may contain short-term jitter, a sliding-window mean filter is applied to the relative motion sequence:

(13) $${\tilde{m}}_{t}=\frac{1}{\left|\Omega_{t}\right|}\sum_{q\in \Omega_{t}}m_{q},$$

where the temporal window is centered at frame t. In this study, the sliding-window size is W = 15. To suppress very small numerical fluctuations during static intervals, smoothed values smaller than epsilon are set to zero; epsilon = 10^−5^ is used in all default experiments.

The smoothed motion magnitude is then normalized to obtain the motion activity coefficient:

(14) $$a_{t}=\frac{{\tilde{m}}_{t}-{\tilde{m}}_{min}}{{\tilde{m}}_{max}-{\tilde{m}}_{min}+10^{-9}},$$

where ${\tilde{m}}_{min}$ and ${\tilde{m}}_{max}$ are the minimum and maximum values of the smoothed motion magnitude in the video sequence. The coefficient $a_{t}$ is constrained to [0, 1], where a larger value indicates stronger wrist-related motion activity.

### 2.5. Adaptive Threshold Generation

The motion activity coefficient $a_{t}$ is used to generate a frame-wise adaptive threshold for keyframe selection. To sample rapid action changes more sensitively, the threshold is adjusted inversely with motion activity. Specifically, higher motion activity leads to a lower threshold, making frames with fast assembly transitions more likely to be selected:

(15) $$T_{t}=T_{max}-a_{t}\left(T_{max}-T_{min}\right),$$

where $T_{min}$ and $T_{max}$ denote the lower and upper bounds of the adaptive threshold, respectively. Under this mapping, a larger motion activity coefficient produces a lower threshold, making keyframe selection more sensitive during rapid assembly motion. Conversely, during low-activity intervals, the threshold becomes higher, which helps reduce redundant keyframe selection caused by minor pose fluctuations.

For a specified output budget, $T_{min}$ is determined by a 30-iteration binary search over [0.01, 3.0], and $T_{max}$ is set to 2.5 $T_{min}$. At each iteration, the complete sequential selection rule is executed and the threshold pair whose native output count is closest to the target is retained. Unless otherwise stated, the reference budget is 271 keyframes per segment. Exact-budget comparisons apply the deterministic capacity-calibration rule described in Section 3.4, whereas native-output tables report the uncorrected sequential result.

### 2.6. Keyframe Selection and Reference Feature Update

After the adaptive threshold is obtained, each frame is compared with the current reference keyframe in the skeleton feature space. The first frame is selected as the initial keyframe, and its feature vector is used as the initial reference feature. For frame t, the feature distance is calculated as:

(16) $$d_{t}={\left\|f_{t}-f_{ref}\right\|}_{2},$$

where $f_{ref}$ denotes the skeleton feature vector of the most recently selected keyframe. A frame is selected as a new keyframe when either of the following conditions is satisfied:

(17) $$d_{t}\geq T_{t}\;t-k_{last}>G_{max},$$

where the reference index denotes the most recently selected keyframe and $G_{max}$ is the maximum allowed frame gap between adjacent keyframes. In this study, $G_{max}$ is set to 50. Once frame t is selected, it is added to the keyframe index set and its skeleton feature becomes the new reference feature. This incremental update ensures that each subsequent decision is made relative to the latest selected keyframe.

The final output of the proposed method is the keyframe index set:

(18) $$K=\left\{k_{1},k_{2},\ldots ,k_{M}\right\},$$

where M is the number of selected keyframes. The selected keyframes retain representative skeleton-motion information while reducing temporal redundancy in the original industrial assembly videos.

### 2.7. Algorithm Summary and Parameter Settings

To improve reproducibility, the detailed execution procedure of the proposed method is summarized in Table 2. The line numbers in Table 2 describe the algorithmic operations and are independent of the five-stage workflow shown in Figure 1.

The default parameter settings are summarized in Table 3. $T_{min}$ is searched over [0.01, 3.0] for 30 iterations under the specified keyframe budget, and $T_{max}$ = 2.5 $T_{min}$. The neck is the default anchor; the hip center is used only for the anchor-comparison experiment.

## 3. Results

### 3.1. Experimental Setup and Dataset

The experiments used three temporally non-overlapping industrial electronics-assembly video segments (Group-1, Group-2, and Group-3) extracted from one longer continuous recording captured at the same workstation. The three segments contain different operation content and distinct pose trajectories. They are treated as separate file-level test cases for evaluating within-recording variation, but not as independent recording-level replications because they share the same camera, workstation, general operator setting, and acquisition environment. The operations include material retrieval and placement, label or material preparation, component positioning, cable-related manipulation, fine manual assembly, and workpiece transfer. Table 4 reports duration, frame rate, resolution, frame count, motion-intensity category, and operation characteristics for each analyzed segment.

Each segment contains one primary operator for pose-based analysis. The decoded videos contain 2882, 2938, and 3207 frames for Group-1, Group-2, and Group-3, respectively. The Group-1 OpenPose table contains 2881 usable skeleton records because the final decoded video frame has no corresponding stored pose record; all Group-1 skeleton-based experiments therefore use the common aligned length of 2881 frames. Group-2 and Group-3 contain 2938 and 3207 aligned skeleton records. The segments differ in motion amplitude, operation frequency, side-reaching behavior, and keypoint reliability; these differences are considered when interpreting metric variation.

The three analyzed segments are temporally non-overlapping and contain different operation content, but they originate from one industrial assembly-line monitoring recording. They share the same fixed monocular camera, workstation, recording environment, and general operator setting. The fixed-view design reflects the intended low-cost monitoring scenario, but the segment-level results do not constitute cross-recording, cross-operator, cross-camera, or cross-site evidence. The elevated oblique camera observes the work surface and the operator’s upper body, while partial occlusion occurs when hands or arms overlap the workpiece, workstation structures obscure joints, or the operator bends and reaches toward side material areas. The dataset is therefore a single-recording case study with three distinct segment-level test cases. Experiments using additional viewpoints and independently recorded industrial videos were not feasible within the resources available for the present study. Extending the experiment to additional cameras and viewpoints would substantially increase the combined costs of video acquisition, storage, backup, transfer, computation, preprocessing, and manual annotation. These resource and cost constraints explain why the present dataset remains bounded, but they do not remove the need for future external validation using independently recorded data. No face images, facial features, identity labels, personal identity information, or biometric identifiers are retained in the derived research dataset reported here, and no identity analysis is performed.

Table 5 summarizes the experimental environments used in this study. Keyframe extraction and post-processing were performed on a local Windows workstation, where OpenPose BODY-25 skeleton data were used as the input representation. To characterize end-to-end computational cost, an additional runtime benchmark was conducted in an AutoDL Linux container equipped with an NVIDIA GeForce RTX 4090D GPU. The selected joints, confidence filtering strategy, interpolation rules, and keyframe update procedure are reported in Section 2.7 and Table 3.

### 3.2. Evaluation Metrics

The evaluation uses six complementary trajectory- and keyframe-level metrics to describe compression efficiency, trajectory reconstruction, temporal coverage, sampling stability, redundancy, and semantic separation. In addition, Event Recall and Mean Temporal Error are introduced for the manually annotated assembly-event evaluation. Together, these metrics assess low-level motion preservation and high-level assembly-event coverage.

Compression Ratio (*CR*): *CR* measures the proportion of frames removed from the original video. A higher value indicates a greater reduction in temporal redundancy.

(19) $$CR=\left(1-\frac{N_{key}}{N_{total}}\right)\times 100\%,$$

where $N_{key}$ and $N_{total}$ denote the numbers of selected keyframes and original video frames, respectively.

Trajectory Reconstruction Error (*Etr*): *Etr* is the root mean square error between the original skeleton trajectory and the trajectory reconstructed by linear interpolation from the selected keyframes. A lower value indicates better trajectory reconstruction.

(20) $$E_{tr}=\sqrt{\frac{1}{N_{total}\cdot K}\sum_{t=1}^{N_{total}}\sum_{j=1}^{K}\|P_{t,j}-{\hat{P}}_{t,j}{\|}^{2}},$$

where K is the number of evaluated joints, and the original and reconstructed joint coordinates are compared over the video sequence.

Trajectory Coverage (*TC*): *TC* measures the normalized preservation of the original skeleton trajectory range. A higher value indicates that the selected keyframes retain a larger proportion of the observed motion trajectory.

(21) $$TC=\left(1-\frac{E_{tr}}{\Delta P_{max}}\right)\times 100\%,$$

The normalization term is the maximum geometric range of the original trajectory in the evaluated feature space.

*TC* is derived from the same reconstructed skeleton trajectory used to calculate Etr and is therefore reported as a complementary normalized description rather than as an independent validation criterion. *Etr* and *TC* should be interpreted jointly.

Coefficient of Variation in Semantic Distance (*CVsd*): *CVsd* measures the relative variation in semantic distances between adjacent selected keyframes. A lower value indicates more stable semantic increments between neighboring keyframes.

(22) $$CV_{sd}=\frac{\sigma (D)}{\mu (D)},$$

The metric is calculated from the standard deviation and mean of the adjacent-keyframe semantic distances.

Keyframe Redundancy (*KR*): *KR* estimates semantic similarity between adjacent keyframes using a Gaussian function. For each evaluated sequence, the Gaussian bandwidth sigma is set to that sequence’s mean adjacent-keyframe semantic distance. A lower *KR* indicates less semantic overlap. This scale-normalized definition is applied identically to every method.

(23) $$KR=\frac{1}{N_{key}-1}\sum_{i=1}^{N_{key}-1}\exp\left(-\frac{\|f_{i}-f_{i+1}{\|}^{2}}{\sigma^{2}}\right),$$

Because σ is derived by the same rule for each output sequence, *KR* reflects the shape of the adjacent-distance distribution rather than an absolute feature-space scale.

Mean Semantic Distance (*MSD*): *MSD* is the mean skeleton feature distance between adjacent selected keyframes. A higher value indicates greater separation between neighboring keyframes, but it must be interpreted together with *Etr*, *TC*, and the number of selected frames.

(24) $$MSD=\frac{1}{N_{key}-1}\sum_{i=1}^{N_{key}-1}\left\|f_{i}-f_{i+1}\right\|,$$

*Etr*, *TC*, *CVsd*, *KR*, and *MSD* are calculated from interpolated trajectories in normalized skeleton space. This common representation enables internally consistent pose-trajectory analysis, but it is aligned with the proposed selection feature and may favor a skeleton-based selector. Moreover, interpolation can smooth long missing-keypoint intervals and may artificially improve apparent semantic regularity. Accordingly, these measures are reported as representation-specific diagnostics and are not treated as independent evidence of practical superiority.

Event Recall (*ER*): *ER* is the proportion of manually annotated assembly-event boundaries that have at least one selected keyframe within a predefined temporal tolerance. One-to-one matching is used so that a selected keyframe cannot match more than one annotated event. ER is calculated as $ER=\frac{N_{match}}{N_{event}}\times 100\%$, where $N_{match}$ is the number of matched events and $N_{event}$ is the total number of manually annotated events.

Mean Temporal Error (*MTE*): *MTE* is the mean absolute temporal distance between each matched manual event and its nearest matched keyframe. A lower MTE indicates that the selected keyframes are located closer to the annotated process transitions. Unmatched events are included in ER but are excluded from the MTE average because no temporal correspondence is available.

Statistical analysis: The keyframe-selection implementations are deterministic for fixed inputs and parameters; repeated execution on the same file therefore does not constitute an additional experimental trial. The three analyzed files are temporally non-overlapping segments containing different operation content and distinct pose trajectories. They provide three valid file-level test cases for assessing within-recording variation. Trajectory and semantic metrics are summarized across these files using means and sample standard deviations. Because the files originate from the same continuous recording and share the same camera, workstation, general operator setting, and acquisition environment, the analysis is descriptive at both the file and event levels and does not treat the files or temporally nested transitions as independent population-level observations. Annotation agreement is quantified using chronology-preserving one-to-one boundary matching across the three files, and event recall and temporal errors characterize transition coverage and localization within the evaluated recording. Random degradation experiments are summarized across perturbation seeds as controlled robustness tests rather than as independent industrial recordings.

### 3.3. Results on Three Industrial Assembly Video Segments

**Figure 2:**
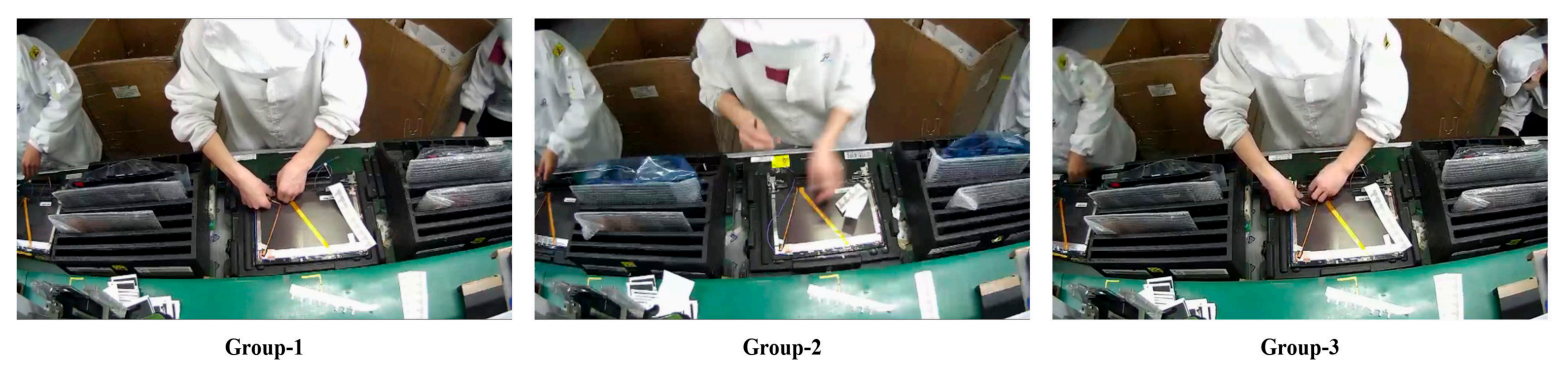
Representative frames from Group-1, Group-2, and Group-3 industrial assembly videos.

The proposed method with the default neck anchor was evaluated separately on the three temporally non-overlapping video segments. Figure 2 shows representative frames, and Table 6 reports the native sequential output for each segment. These are three distinct file-level test cases within the shared recording environment; they are not interpreted as three independent recording-level replications.

With the reference output budget set to approximately 271 frames per segment, the method selects 271, 270, and 271 keyframes from Group-1, Group-2, and Group-3, corresponding to frame-reduction ratios of 90.59%, 90.81%, and 91.55%. Group-2 obtains the lowest *Etr* (0.0604) and highest *TC* (93.93%), whereas Group-1 has the largest *MSD* (0.8877). These differences reflect segment-specific pose amplitudes, temporal organization, and keypoint reliability and should not be interpreted as a ranking of the three segments.

Across the three segments, *CVsd* ranges from 0.2289 to 0.4467 and *KR* remains close to 0.60 under the scale-normalized definition. These results establish the native behavior of the final method configuration in the shared recording environment; they do not establish cross-camera, cross-operator, or cross-site generalization.

### 3.4. Comparative Experiments

#### 3.4.1. Baseline Methods and Matched-Budget Protocol

The comparison includes uniform sampling, pose-space clustering, pixel-space clustering, a fixed-threshold pixel-MSE method, ORB + Flow, motion-adaptive sampling, and a modern pretrained video-transformer baseline. ORB + Flow combines ORB feature matching and Lucas-Kanade optical flow [22,23]. The clustering-based implementations use k-means++ initialization to improve representative-center selection [24]. The transformer baseline uses MViT-V2-S pretrained on Kinetics-400 without fine-tuning [25]. For each segment, 96 uniformly distributed clip centers are used. Each clip contains 16 frames sampled with a temporal stride of three frames; the official preprocessing resizes the shorter spatial side to 256 pixels, applies a 224 × 224 center crop, and normalizes RGB values using the pretrained-weight configuration. The resulting 768-dimensional clip features are L2-normalized, linearly interpolated over the complete frame timeline, and clustered to select exactly 60, 120, or 271 representative frame centers. Uniform sampling provides temporal coverage, pose- and pixel-space clustering select cluster representatives, fixed-threshold selection ranks pixel-MSE changes, and motion-adaptive sampling allocates frames from pixel activity. All image-based features are computed from decoded video frames.

All methods are constrained to the same output budget. The pretrained MViT baseline adds a modern transformer representation without task-specific training on the analyzed industrial videos, while the proposed method and the other baselines remain deterministic after their parameters are fixed. The MViT setting is a feature-clustering baseline rather than an end-to-end supervised summarization system; transformer/Mamba summarization and action-segmentation networks, including representative transformer and multi-stage temporal-convolution approaches [26,27], require task-specific labels or use different output objectives and therefore remain outside the direct comparison. Consequently, the results compare the proposed method with both conventional methods and one pretrained modern video representation, but they do not establish superiority over every possible learned video-selection architecture.

For the capacity-controlled comparison, every method selects exactly 271 frames from each analyzed file. The proposed method uses the default neck-anchor configuration, $c_{min}$ = 0.2, W = 15, $G_{max}$ = 50, bilateral wrists, eight upper-body feature joints, and inverse activity-threshold mapping. A 30-iteration binary search stores the native output with the smallest absolute count error, breaking equal errors by the earlier search iteration. If the stored count is below the budget, unselected frames are ranked by |$d_{t}\;$ − $T_{t}$| in ascending order and then by frame index; frames are added in that order until the target is reached. If the count is above the budget, non-endpoint selected frames are ranked by the sum of their distances to the preceding and following selected frames in ascending order and then by frame index; a frame is removed only if the resulting temporal gap does not exceed $G_{max}$. Capacity correction does not rerun the sequential reference updates. In the present 271-frame comparison, only Group-2 requires one addition (frame 790); Group-1 and Group-3 already contain 271 frames. The matched-budget comparison results are summarized in Table 7.

#### 3.4.2. Matched-Budget Comparative Results

**Figure 3:**
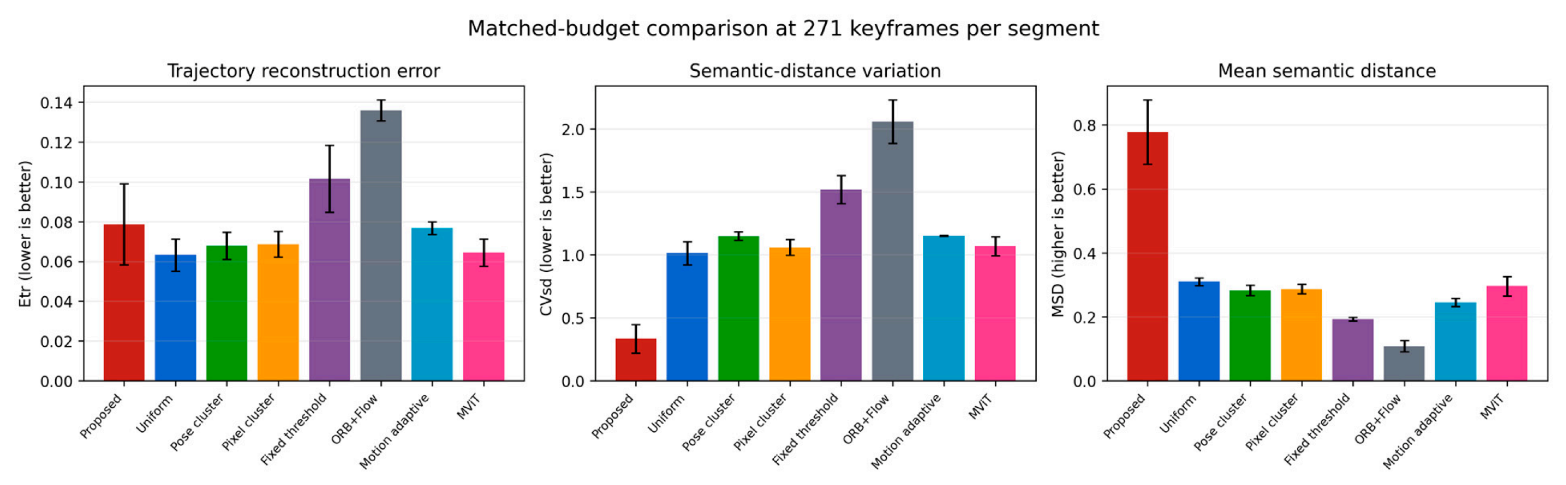
Matched-budget comparison of *Etr*, *CVsd*, and *MSD* for eight methods at 271 keyframes per segment. Bars show the mean across three video segments, and error bars show one standard deviation.

Figure 3 compares trajectory reconstruction and semantic-spacing metrics under the matched 271-frame budget. The proposed method obtains *CVsd* = 0.3340 ± 0.1117 and *MSD* = 0.7781 ± 0.1003. The pretrained MViT baseline obtains lower *Etr* (0.0645 ± 0.0068) and higher *TC* (93.48% ± 0.74%), but its *CVsd* is 1.0681 ± 0.0754 and its *MSD* is 0.2961 ± 0.0305. Thus, MViT feature clustering improves trajectory coverage while the proposed method retains substantially more regular and more widely separated pose-semantic samples. This result indicates a metric-specific advantage rather than universal superiority over the transformer representation.

**Figure 4:**
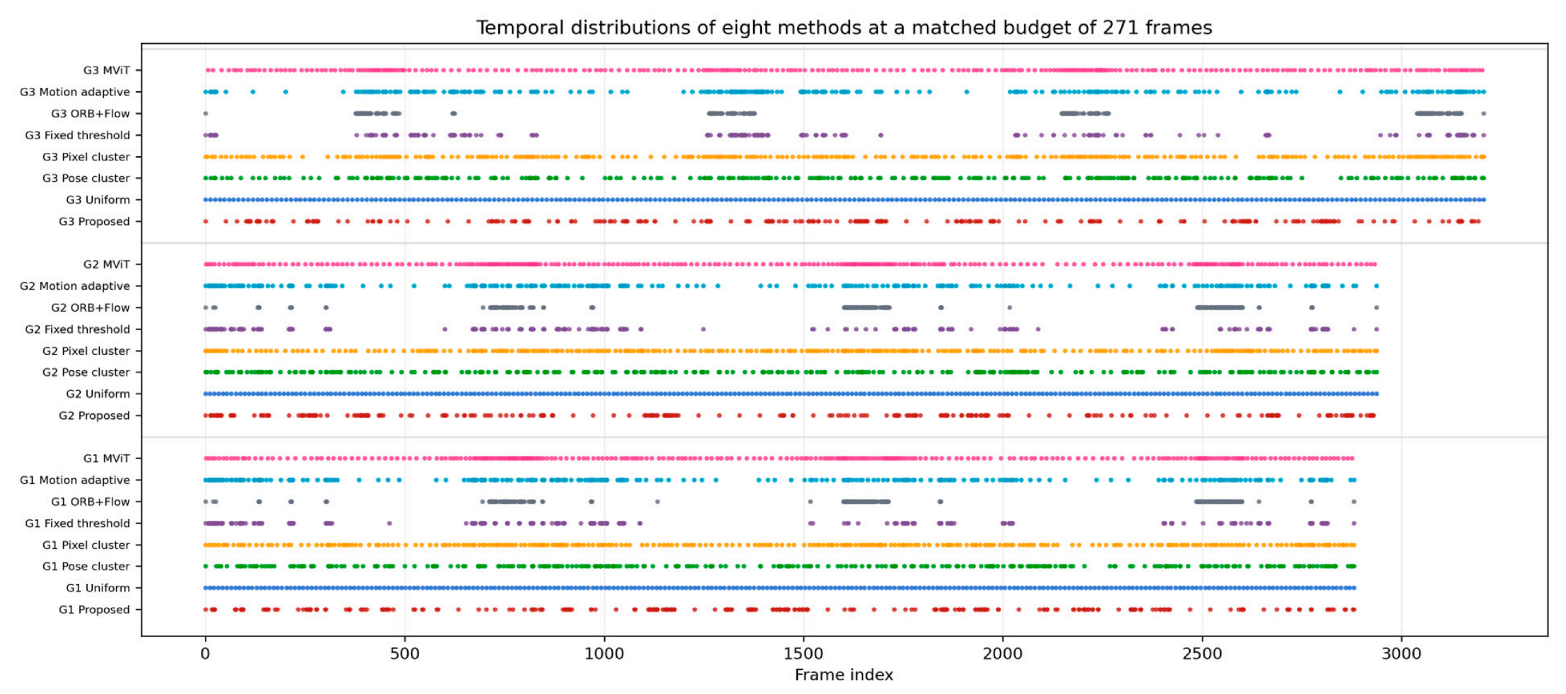
Temporal distributions of keyframes selected by eight methods at a matched budget of 271 frames per video segment.

Figure 4 visualizes the temporal locations selected by all eight methods at the same budget. The proposed method distributes frames according to sequential skeleton change and the maximum-gap constraint, whereas MViT and the other clustering or motion methods produce different local concentration patterns. Because every row contains 271 frames, the temporal patterns can be compared without output-size confounding.

**Figure 5:**
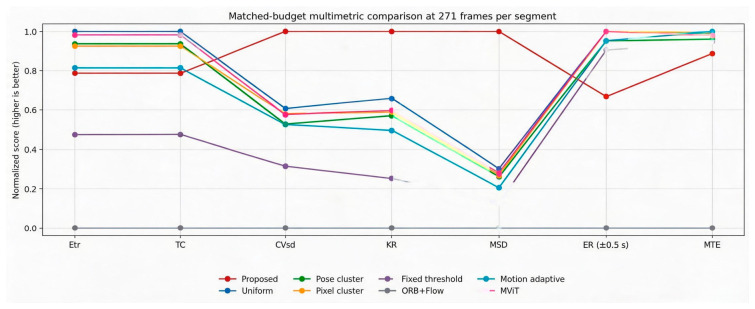
Normalized multimetric comparison of eight methods at 271 keyframes per segment. Each metric is oriented so that a higher normalized score indicates a more favorable value.

Figure 5 combines five normalized skeleton-trajectory metrics with process-transition recall and temporal error. The proposed method has the most favorable *CVsd*, *KR*, and *MSD* values, but this reflects its direct optimization in skeleton space. Uniform sampling, pixel-space clustering, motion-adaptive sampling, and MViT provide stronger process-transition coverage or lower reconstruction error. The figure therefore demonstrates different objective preferences rather than an overall ranking of keyframe-extraction quality.

#### 3.4.3. Multi-Budget Process-Transition Comparison

**Figure 6:**
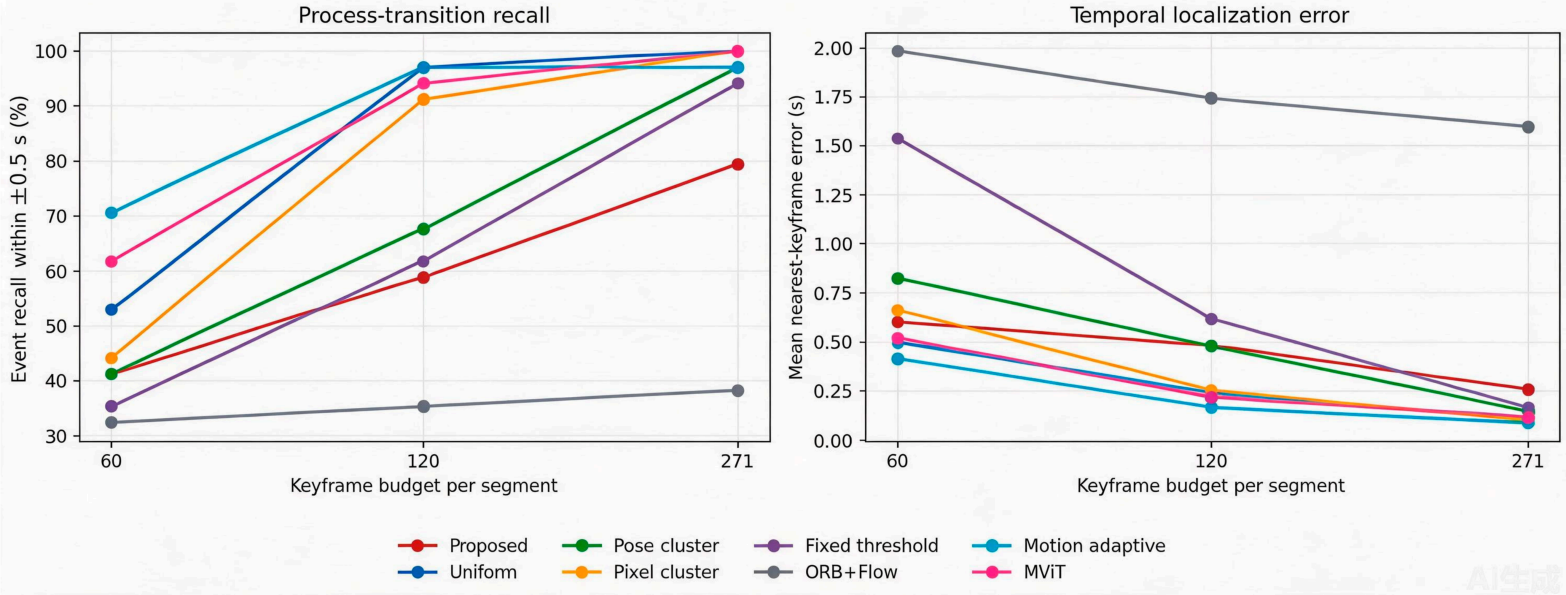
Event recall within ±0.5 s and mean nearest-keyframe temporal error for eight methods under matched keyframe budgets of 60, 120, and 271 frames per segment. Each point aggregates the 34 manually annotated transitions from the three video segments.

The same eight implementations were evaluated at 60, 120, and 271 keyframes per segment. Table 8 reports descriptive event recall within ±0.5 s and mean nearest-keyframe error against the adjudicated 34-event consensus reference. These event outcomes are not used for population-level inference because the events come from temporally dependent segments of one recording. Table 8 summarizes the event-recall and mean nearest-keyframe temporal-error results for the eight methods under the matched keyframe budgets. Figure 6 presents these results for budgets of 60, 120, and 271 frames per segment.

The event-based evaluation addresses process-transition coverage, which is distinct from the pose-state-diversity objective for which the proposed method was designed. At 271 frames per segment, the proposed method retains 27/34 transitions (79.41%), whereas uniform sampling, pixel-space clustering, and MViT retain 34/34 (100%), and motion-adaptive and pose-space clustering each retain 33/34 (97.06%). These results indicate that the proposed method should not be used as a stand-alone event-boundary sampler when transition recall is the primary requirement. Its engineering contribution instead lies in deterministic, training-free, budget-controllable sampling of differentiated operator-pose states, as quantified by the matched-budget skeleton-space diagnostics. The two evaluation views therefore identify complementary operating objectives rather than rendering one of them practically meaningless.

At the 271-frame budget, the proposed method has a descriptive mean nearest-keyframe error of 0.259 s. Uniform sampling, pixel-space clustering, motion-adaptive sampling, and MViT obtain lower errors of 0.107, 0.102, 0.089, and 0.120 s, respectively. No bootstrap interval or event-wise significance test is reported, because treating transitions from the same recording as independent observations would overstate statistical support.

The event comparison and skeleton-space metrics answer different questions. At the 271-frame budget, the proposed selector retains 79.41% of the annotated transitions, while the stronger event-coverage baselines retain 97.06–100%. Conversely, relative to MViT in the common normalized skeleton evaluation, it reduces semantic-distance variation by 68.7% and increases mean adjacent pose separation by 162.8%. This is an objective-dependent trade-off rather than an overall ranking: the proposed selector organizes differentiated pose states and regular skeleton-semantic increments, whereas several temporal, appearance-based, or pretrained baselines retain more annotated process transitions. Consequently, the present evidence supports interpretable, training-free, budget-controllable skeleton-semantic spacing within the evaluated recording; downstream action recognition, process monitoring, or human assessment remains a future validation question.

### 3.5. Ablation Study

#### 3.5.1. Matched-Budget Ablation Protocol

**Figure 7:**
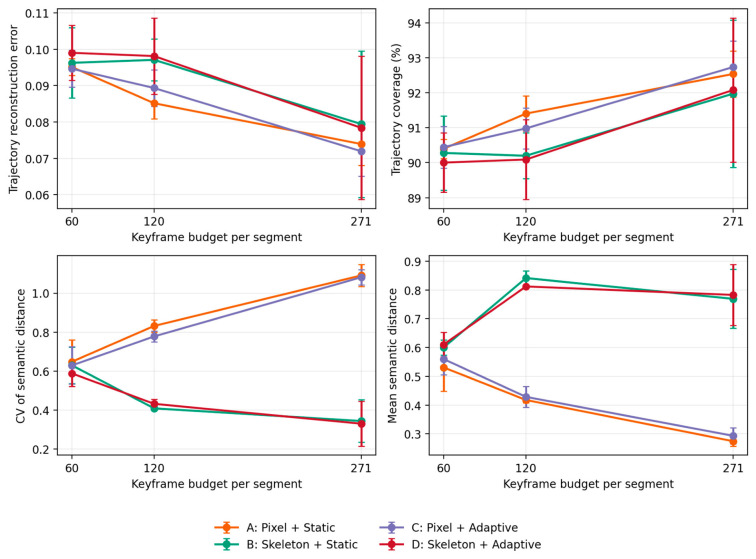
Matched-budget ablation results for *Etr*, *TC*, *CVsd*, and *MSD*. Markers show the mean over the three video segments, and error bars show one standard deviation.

A controlled 2 × 2 ablation separates the effects of feature representation and threshold strategy under matched budgets of 60, 120, and 271 frames per segment. All variants use the same maximum-gap setting and evaluation space. Because the output count is explicitly matched, the analysis focuses on how the two components affect pose-space organization and temporal allocation at each budget. The corresponding results are summarized in Table 9. The corresponding metric trends are shown in Figure 7.

Feature representation produces the largest and most consistent differences. At 271 frames, the skeleton variants obtain *CVsd* values of 0.3439 and 0.3301, compared with 1.0907 and 1.0815 for the pixel variants, while also producing substantially larger *MSD* values. A similar representation-level separation is observed at 120 frames. These results identify the skeleton representation as the primary contributor to the observed semantic-spacing characteristics.

The effect of adaptive thresholding is more budget-dependent. At 271 frames, Skeleton + Adaptive provides modest improvements in *CVsd* and *MSD* over Skeleton + Static. At 120 frames, the static skeleton variant obtains slightly more favorable values across the four reported metrics, while at 60 frames the differences in *CVsd* and *MSD* remain modest and *Etr* and *TC* favor the static variant. These results indicate that adaptive thresholding acts mainly as an activity-aware temporal density-control mechanism rather than as a component expected to improve every global metric under every fixed budget.

At the 60-frame budget, the maximum-gap rule operates close to the target capacity and strongly constrains all variants, reducing the observable effect of threshold adaptation. More generally, exact budget matching controls output-size confounding but also limits the extent to which the adaptive mechanism can express its native frame-density adjustment. The ablation therefore supports a complementary interpretation: skeleton representation determines the principal semantic organization of the selected frames, whereas adaptive thresholding provides secondary, budget-dependent control over their temporal allocation.

#### 3.5.2. Parameter Sensitivity Analysis

**Figure 8:**
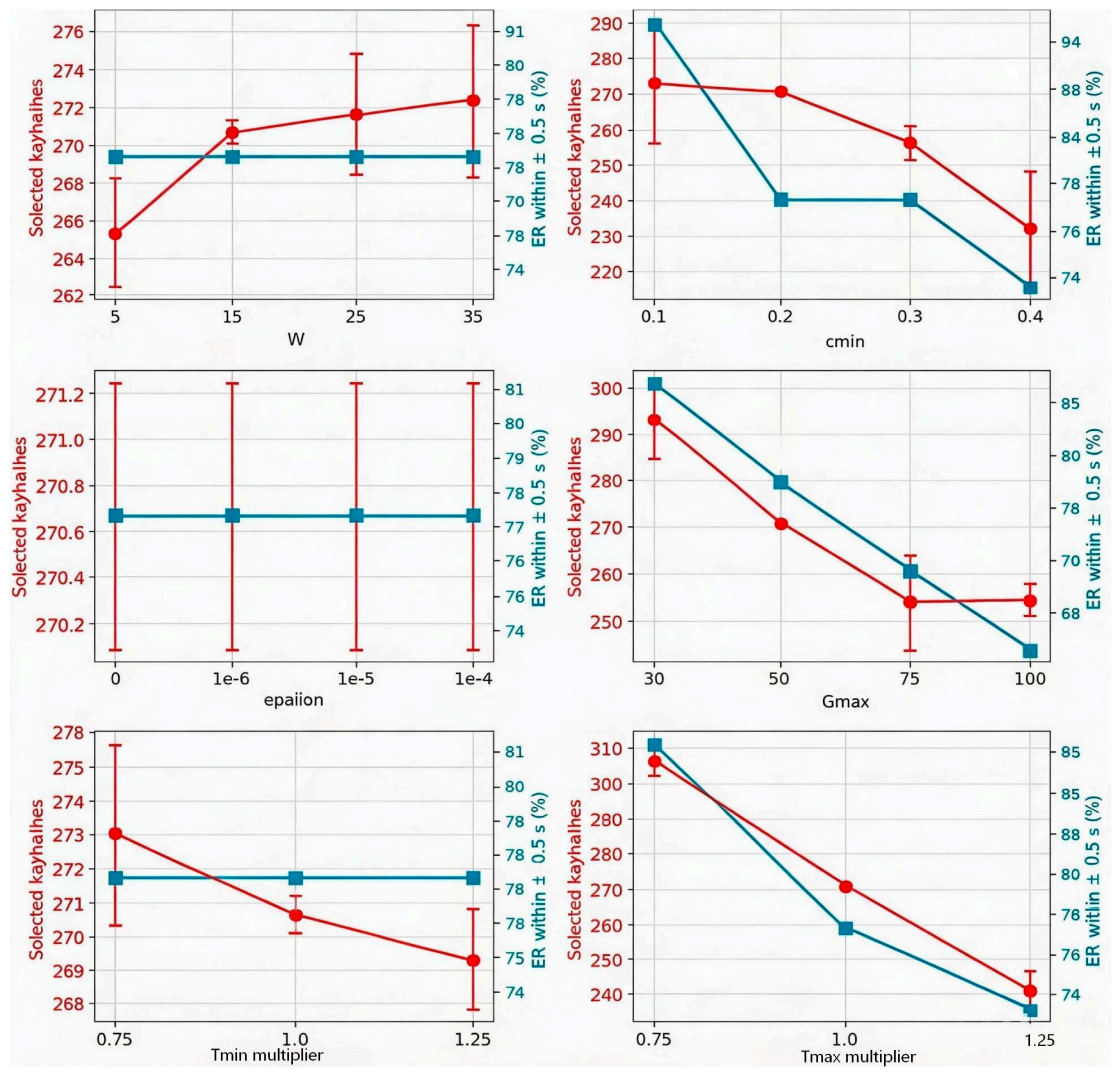
Sensitivity of the selected-frame count and manually annotated event recall to W, $c_{min}$, epsilon, $G_{max}$, the $T_{min}$ multiplier, and the $T_{max}$ multiplier. Error bars show one standard deviation across the three video segments.

A one-factor-at-a-time sensitivity analysis was conducted for W, $c_{min}$, epsilon, $G_{max}$, $T_{min}$, and $T_{max}$. For each segment, the baseline threshold pair was first determined using the default 271-frame configuration. Each parameter was then varied while the remaining parameters and the reference threshold pair were held fixed; thresholds were not retuned after each variation. Multipliers of 0.75, 1.00, and 1.25 were applied separately to $T_{min}$ and $T_{max}$. The results report the mean and standard deviation across the three segments together with event recall within ±0.5 s. The parameter sensitivity trends are shown in Figure 8, and the corresponding numerical results are summarized in Table 10.

The method is relatively insensitive to *W*, epsilon, and $T_{min}$ within the evaluated ranges. Increasing W from 5 to 35 changes the mean output from 265.3 to 272.3 keyframes, while mean event recall remains 77.31%. Changing epsilon from 0 to 10^−4^ produces no measurable change because the affected activity values are already negligible after normalization. $T_{min}$ multipliers from 0.75 to 1.25 change the mean output by fewer than four frames and leave event recall unchanged.

In contrast, $c_{min}$, $G_{max}$, and $T_{max}$ directly affect sampling density. Raising $c_{min}$ from 0.2 to 0.4 reduces the mean output from 270.7 to 232.0 keyframes and decreases event recall from 77.31% to 73.61%, because more low-confidence joints are replaced by interpolated trajectories. Reducing $G_{max}$ from 50 to 30 increases the output to 293.0 frames and improves event recall to 86.81%, whereas increasing $G_{max}$ to 100 reduces event recall to 61.57%. Similarly, decreasing the $T_{max}$ multiplier to 0.75 produces 306.7 keyframes and 86.34% event recall, while increasing it to 1.25 produces 241.0 keyframes and 73.15% recall.

The selected values W=15, $c_{min}$ = 0.2, epsilon = 10^−5^, $G_{max}$ = 50, and $T_{max}$ = 2.5 $T_{min}$ provide an effective balance between compression, semantic separation, and event-boundary coverage for the evaluated assembly sequences. The analysis identifies $G_{max}$ and $T_{max}$ as the parameters requiring the most careful retuning when the desired keyframe budget or action tempo changes.

### 3.6. Anchor-Configuration Results

**Figure 9:**
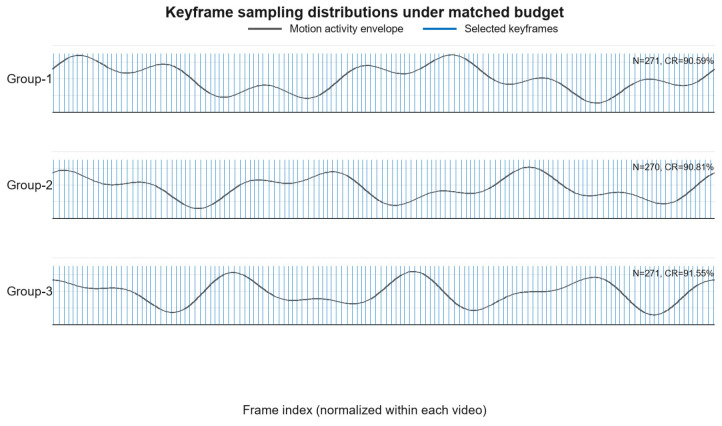
Distribution of selected keyframes under the matched-budget setting across the three video groups.

**Figure 10:**
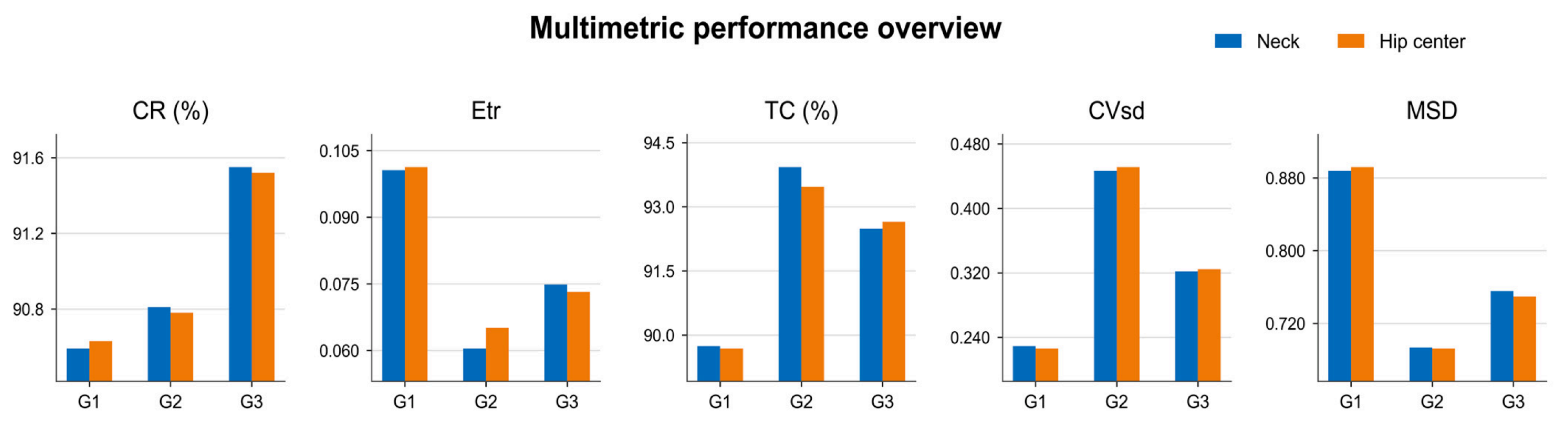
Multimetric comparison of neck-anchor and hip-center-anchor results across the three video groups.

The neck anchor is used by default in the proposed method, while the hip center is evaluated as an alternative under the same parameter settings and an approximately matched 271-frame target. Table 11 reports both configurations on all three segments, thereby isolating anchor replacement without changing the feature joints, motion joints, threshold mapping, or maximum-gap rule. Because no independently annotated physical motion-intensity signal is available, the comparison does not treat either anchor as ground truth; Section 3.8 instead quantifies the discrepancy between their normalized activity estimates and the resulting keyframe sequences. The distribution of selected keyframes under the matched-budget setting is shown in Figure 9. The multimetric comparison of the neck-anchor and hip-center-anchor results is shown in Figure 10.

Both anchor configurations return 270–272 keyframes per segment and compression ratios above 90%. The neck-anchor rows in Table 11 are the same default-method outputs reported in Table 6; the hip-center rows differ only in the anchor used for relative wrist motion.

### 3.7. Manual Assembly-Event Evaluation

The first event set was produced by one human annotator who reviewed each file chronologically and marked the first frame that clearly exhibited a new sustained process phase. Transitions included changes among sustained assembly, material or label handling, component retrieval, workpiece positioning, and workstation transfer. Small adjustments within the same phase were excluded, and candidate boundaries were refined at 0.25 s resolution.

The first human annotation contained 34 file-level transitions: nine in Group-1, nine in Group-2, and 16 in Group-3. A second human annotator independently reviewed the videos using the same written phase definitions and exclusion rules without access to the first timestamps or keyframe outputs. The second set contains 37 candidate transitions: nine, nine, and 19, including three Group-3 boundaries marked uncertain before adjudication. The second annotator’s raw decisions were preserved before author review. Because the three segments are temporally non-overlapping and contain different operation content, agreement is reported across all three file-level annotation sets.

Chronology-preserving one-to-one matching was used for the pre-adjudication reliability analysis. Matches were maximized within each file and total absolute timing error was then minimized. Across all three files, 30 boundaries match within ±0.5 s (precision = 81.08%, recall = 88.24%, F1 = 84.51%, mean absolute difference = 0.092 s), and 32 match within ±1.0 s (precision = 86.49%, recall = 94.12%, F1 = 90.14%, mean absolute difference = 0.133 s). These values describe file-level annotation agreement within the shared recording environment.

Requiring both temporal proximity and the same normalized before/after phase pair gives pooled file-level F1 = 76.06% at ±0.5 s and 81.69% at ±1.0 s. Most remaining disagreements occur in Group-3, where a compound material-handling, workstation-transfer, and positioning episode can be represented either as one coarse process boundary or as several consecutive subphase boundaries.

After the pre-adjudication statistics were frozen, the corresponding author reviewed every unmatched or phase-discordant candidate at 0.25 s resolution using the written event definition and without consulting any keyframe output. Boundaries describing the same sustained transition within ±1.0 s were merged at the first clearly visible new phase. Second-set-only subdivisions were excluded when they represented brief intermediate movement rather than a sustained phase; first-set-only boundaries were retained when the review showed a sustained transition; and phase-discordant temporal matches were resolved from the video. All retain, merge, and exclude decisions were documented during adjudication. This procedure yields a 34-transition consensus reference whose timestamps coincide with the original 34-event evaluation reference, so Table 8 and Table 12 do not require retrospective numerical changes. Although the final consensus timestamps coincide with the first annotation set, this outcome was obtained only after independent second annotation and review of all unmatched or phase-discordant candidates; the first annotation set was not automatically treated as the reference standard. Table 12 presents the event-level comparison between the default neck-anchor method and the hip-center variant against the adjudicated 34-transition consensus reference.

For keyframe evaluation, each consensus event is matched to the nearest unused selected frame within ±0.5 s or ±1.0 s. Event precision is not reported for the extraction algorithms because selected representative frames are not event predictions. The independent annotation audit instead reports boundary-set precision, recall, and F1 between annotators.

The reliability audit shows substantial temporal-boundary correspondence, but agreement decreases when exact phase pairs are required. This pattern reflects ambiguity in the granularity of compound industrial operations. The adjudicated reference improves transparency of the event evaluation but does not overcome the modest number of transitions or the dependence created by one source recording.

### 3.8. Anchor-Selection Analysis

**Figure 11:**
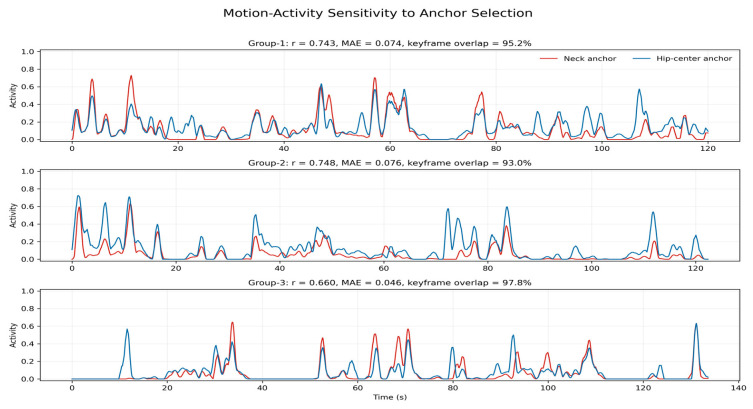
Normalized bilateral-wrist motion-activity sequences obtained with the neck and hip-center anchors. Curves are smoothed over 24 frames for visualization only; all statistics and keyframe decisions use the original activity coefficients. Each panel reports the activity correlation, activity MAE, and keyframe overlap within ±3 frames.

The neck joint may move during bending, head turning, or postural changes and is therefore not considered a universally stable anchor. Conversely, the hip center can be unreliable when the lower torso is obscured by the workstation. Table 13 compares the normalized activity coefficients produced by the two anchors using Pearson correlation, mean absolute discrepancy (MAE), root mean square discrepancy (RMSE), the 95th percentile of absolute discrepancy, overlap between the highest 10% activity frames, and keyframe overlap within ±3 frames. These quantities measure anchor sensitivity rather than physical motion-intensity error because no external motion-intensity ground truth is available. The normalized bilateral-wrist motion-activity sequences obtained with the two anchors are shown in Figure 11.

The activity sequences have Pearson correlations of 0.743, 0.748, and 0.660 for Group-1, Group-2, and Group-3, respectively. Their normalized activity MAEs range from 0.0461 to 0.0756, while the 95th-percentile absolute discrepancies range from 0.2465 to 0.3252. The overlap between the highest 10% activity frames is lower, ranging from 39.78–46.63%, showing that the two anchors do not identify all local activity peaks identically. Nevertheless, 92.99–97.79% of the resulting keyframes overlap within ±3 frames, and Table 11 and Table 12 show only small differences in trajectory metrics and event preservation. Anchor selection therefore affects local motion-intensity estimation more strongly than the final keyframe capacity and event-level results under the evaluated configuration.

### 3.9. Controlled Skeleton-Degradation Robustness Analysis

**Figure 12:**
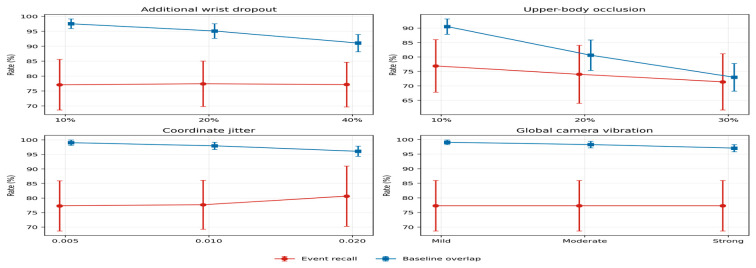
Event recall and temporal overlap with the baseline keyframe set under controlled wrist dropout, upper-body occlusion, coordinate jitter, and global camera vibration. Error bars show one standard deviation over 30 runs per perturbation level.

Controlled perturbation experiments were conducted to quantify sensitivity to pose-estimation failures and global camera motion without claiming cross-scene robustness. Occlusion-aware pose research indicates that joint visibility and structural reasoning are central to reliable pose estimation under partial observation [28]. Four perturbations were evaluated after coordinate normalization: additional dropout of both wrist confidences, contiguous upper-body occlusion affecting the selected feature joints, zero-mean Gaussian coordinate jitter, and frame-wise global camera vibration. The camera-vibration condition applies the same translation and rotation to every joint in a frame, distinguishing it from independent joint jitter. Mild, moderate, and strong levels use normalized horizontal translation amplitudes of 0.005, 0.010, and 0.020 (approximately 3.2, 6.4, and 12.8 pixels at 640-pixel width) combined with rotation amplitudes of 0.25, 0.50, and 1.00 degrees. Each non-baseline condition was repeated with 10 random seeds for each video segment. Baseline thresholds were held fixed, and selected indices were evaluated against the original unperturbed skeleton trajectories. Baseline overlap denotes the proportion of baseline keyframes for which a perturbed-result keyframe occurs within three frames. The controlled skeleton-degradation and global camera-motion results are summarized in Table 14. The event recall and temporal-overlap results under the controlled perturbations are shown in Figure 12.

Additional wrist dropout has a limited effect on event recall within the tested range. At 40% additional dropout, mean event recall remains 77.18%, close to the 77.31% baseline, while the selected-frame count decreases from 270.7 to 248.4 and baseline overlap decreases to 91.07%. This indicates that temporal interpolation and the use of two wrist joints can preserve many coarse process boundaries during intermittent wrist failures, although the sampling density changes.

Contiguous upper-body occlusion has a stronger effect because it simultaneously disrupts the decision feature and motion activity estimation. At 30% occluded frames, the mean output decreases to 206.1 keyframes, event recall decreases to 71.39%, and baseline overlap decreases to 72.98%. Independent coordinate jitter produces smaller changes, with overlap remaining above 96%. The results under simulated global camera vibration are more stable: event recall remains 77.31% at all three levels, while baseline overlap decreases from 99.08% under mild vibration to 97.06% under the strongest combination of approximately 12.8-pixel translation and 1-degree rotation. The relative-wrist activity calculation therefore suppresses much of the simulated frame-wise global motion, although the experiment operates on transformed skeleton coordinates rather than rerunning pose estimation on geometrically transformed videos.

These perturbation experiments quantify robustness to synthetic skeleton degradation and controlled global coordinate transforms only. The camera-motion simulation tests the downstream skeleton representation after a common frame-wise affine transform; it does not measure how OpenPose itself responds to blurred, cropped, or resampled camera-motion video. Background clutter is also not modified in these skeleton-level tests. The end-to-end illumination experiment in Section 3.10 evaluates one image-domain factor, but independently recorded viewpoints, operators, workplaces, real camera motion, background changes, and real OpenPose failure distributions remain outside the current evidence.

### 3.10. Controlled Illumination-Degradation Analysis

Controlled photometric transformations were applied before pose extraction: original lighting, darkening ×0.5, overexposure ×1.5 with clipping, and contrast reduction ×0.6. OpenPose and the complete keyframe pipeline were rerun independently under each condition with unchanged parameters. Because this rerun generated a separate original-lighting skeleton sequence and capacity-calibration path, it is analyzed only as an internal pose-detection and keyframe-stability diagnostic and is not used to replace the unified main comparison in Table 7 and Table 8.

**Figure 13:**
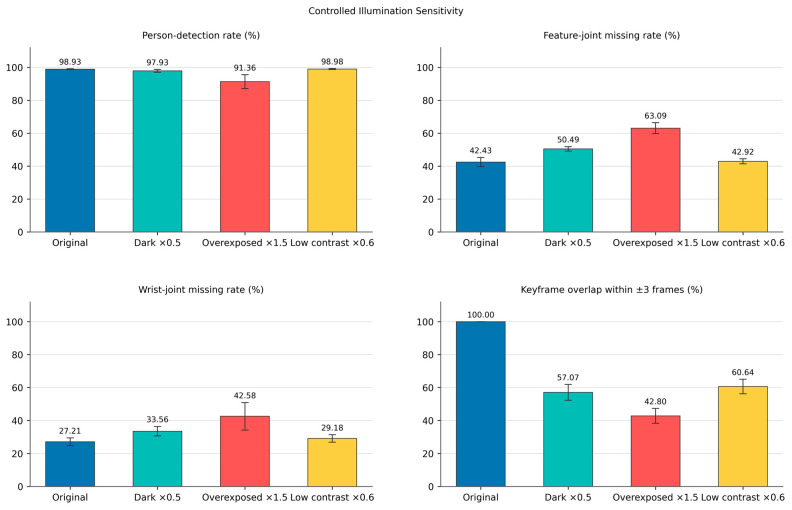
Person-detection rate, feature-joint missing rate, wrist-joint missing rate, and keyframe overlap with the original-lighting rerun under controlled illumination transformations. Error bars show one standard deviation across the three segments.

The illumination analysis reports person-detection rate, feature- and wrist-joint missing rates, and keyframe overlap with the original-lighting rerun within ±3 frames. *Etr*, *CVsd*, *MSD*, and event recall are removed from this table because their rerun-specific original condition is not numerically interchangeable with the main protocol. The internal illumination diagnostics are summarized in Table 15. The controlled illumination sensitivity results are shown in Figure 13.

Low contrast produces the smallest upstream change: detection remains 98.98% and feature-joint missing rate is 42.92%, compared with 98.93% and 42.43% in the original-lighting rerun. Darkening increases feature-joint missing rate to 50.49%. Overexposure has the strongest effect, reducing detection to 91.36% and increasing feature- and wrist-joint missing rates to 63.09% and 42.58%.

Relative to the original-lighting rerun, keyframe overlap within ±3 frames is 57.07% under darkening, 42.80% under overexposure, and 60.64% under low contrast. These results quantify how controlled photometric degradation propagates through pose estimation to the final keyframe decisions. Overexposure produces the largest upstream pose degradation and the greatest change in selected locations, identifying pose-detection quality as an important operating condition of the framework. The analysis therefore provides an internal robustness diagnostic and motivates exposure control, confidence monitoring, or more illumination-robust pose estimation in future implementations. Because the experiment uses transformed versions of the same recordings and does not re-evaluate event recall, external lighting generalization and event preservation remain separate questions for future validation.

### 3.11. Computational Cost and Missing-Keypoint Analysis

**Figure 14:**
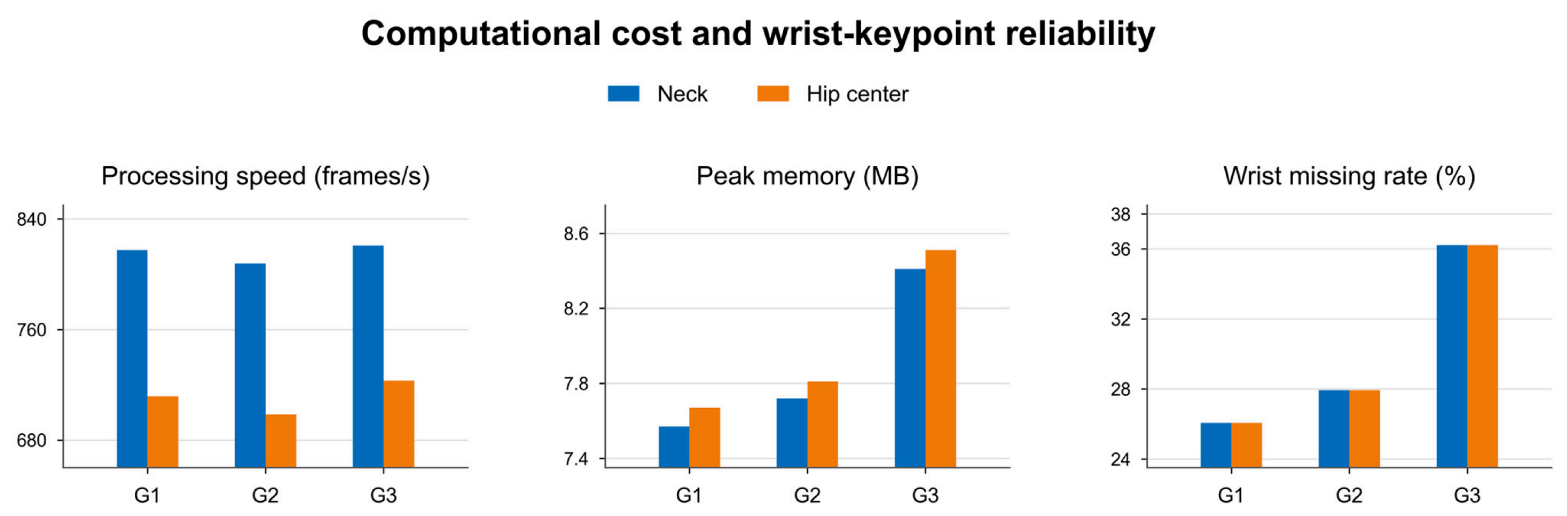
Processing speed, peak memory, and wrist-keypoint missing rate of the decision-stage implementation.

Computational cost was evaluated at two levels. Resource-aware pose-estimation research highlights the need to balance multi-person pose accuracy and computational constraints [29]. Table 16 reports the skeleton-based decision stage after OpenPose inference, while Table 17 reports an end-to-end GPU benchmark that includes video decoding, OpenPose BODY-25 inference, JSON loading, skeleton preprocessing, activity estimation, threshold search, and keyframe selection. The benchmark uses the first 300 frames of each segment and three repeated runs per segment in the AutoDL Linux environment listed in Table 5. The processing speed, peak memory, and wrist-keypoint missing rate of the decision-stage implementation are shown in Figure 14.

**Figure 15:**
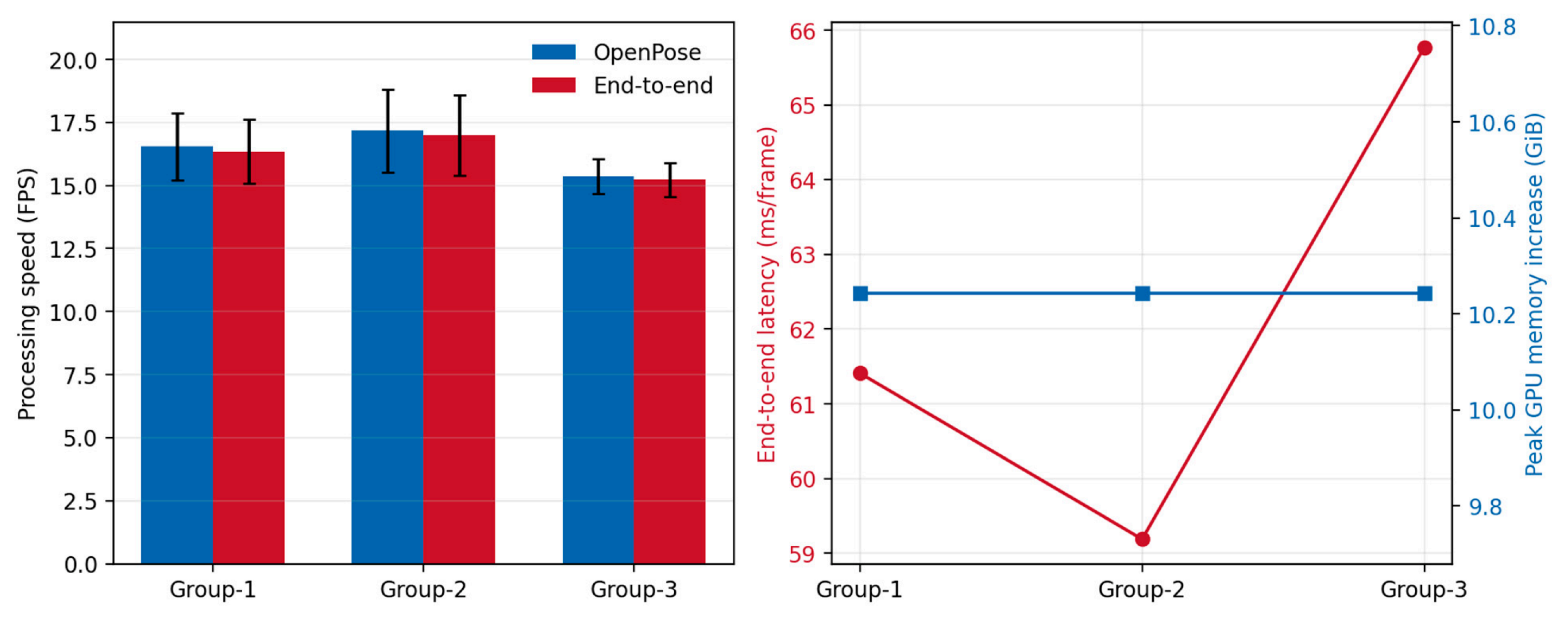
OpenPose inference speed, end-to-end processing speed, average latency, and peak GPU memory increase in the AutoDL benchmark.

After OpenPose keypoints have been obtained, the decision stage processes approximately 699–821 frames/s with measured Python peak memory below 9 MB. Feature-joint missing rates are high, ranging from 33.75% to 50.00%, and wrist-joint missing rates reach 36.22%. Because the primary metrics use temporally interpolated trajectories, Section 3.12 reports interpolation-gap distributions and re-evaluates the matched 271-frame outputs of the proposed method and all seven baselines using observed-joint-only and confidence-weighted metrics. These analyses show that interpolation lowers *Etr* and *CVsd*, while the proposed method’s comparative semantic-spacing pattern persists when missing joints are excluded or downweighted. The magnitude of the semantic-regularity result should therefore be interpreted together with pose confidence, observed-joint coverage, and long missing gaps. The AutoDL benchmark results for OpenPose inference speed, end-to-end processing speed, latency, and peak GPU memory increase are shown in Figure 15.

Across the nine valid GPU runs, OpenPose processes 16.36 ± 1.37 frames/s and the end-to-end pipeline processes 16.19 ± 1.33 frames/s, corresponding to 62.12 ± 4.78 ms per frame. The peak GPU memory increase is 10,489 MB, and the mean valid-person detection rate is 99.56%. Efficient video architectures such as temporal-shift and expanded 3D networks, together with recent masked-autoencoder video models, provide alternative directions for scalable video representation learning [30,31,32], but they do not remove the pose-inference bottleneck measured in the present pipeline. The small difference between OpenPose-only and end-to-end throughput indicates that pose inference dominates runtime, whereas the downstream skeleton-based decision stage adds little overhead. Under the reported CPU configuration, the MViT baseline processes 96 pretrained clips per segment in 148.1–159.5 s before clustering. Because the methods were benchmarked on different hardware, this result is reported as a configuration-specific cost rather than a hardware-normalized comparison.

A CPU-mode validation was also attempted using the OpenPose build used in the experiment and the --num_gpu 0 option. Although the executable returned successfully, all nine CPU runs produced zero detected-person frames and therefore represented empty JSON generation rather than valid BODY-25 inference. The apparent CPU speed was excluded as invalid. Consequently, this study reports verified GPU end-to-end cost and decision-stage CPU cost, but does not claim valid full OpenPose CPU throughput. The measured 16.19 FPS is below the 24 FPS video rate of the evaluated videos, indicating suitability for offline processing, but not strict real-time deployment, on this configuration.

### 3.12. Missing-Keypoint Gap and Matched-Baseline Observed-Joint Sensitivity Analysis

A missing gap is defined as a maximal consecutive run in which one of the eight feature joints has OpenPose confidence below 0.2. Table 18 aggregates gap lengths across the eight joints in each file. Although the median gap is two frames (0.083 s) in every file, the upper tail increases across the files: the 95th percentile is 11, 25, and 33 frames, and the maximum is 155, 150, and 366 frames for Group-1, Group-2, and Group-3, respectively. The proportions longer than 1.0 s are 1.67%, 5.12%, and 7.70%. Thus, the high frame-wise missing rates include many short gaps but also a smaller number of long gaps for which linear interpolation is a strong modeling assumption.

To test whether the comparative semantic-spacing result is driven by interpolation, the matched 271-frame outputs of the proposed method and all seven baselines were reevaluated under the same two missing-data treatments. Observed-joint-only *Etr* compares each reconstructed trajectory with raw normalized coordinates only where the original OpenPose confidence is at least 0.2. For adjacent keyframes, only joints observed at both endpoints are retained, and each distance is normalized to the eight-joint scale. The confidence-weighted form additionally weights each retained joint by its frame confidence for *Etr* and by the smaller endpoint confidence for adjacent-frame distance. Adjacent pairs with no jointly observed feature joint are excluded. Group-2 uses the same one-frame capacity correction (frame 790) specified for the matched-budget comparison, so every method contributes exactly 271 frames per analyzed file. The matched-baseline missing-data sensitivity results are summarized in Table 19.

The comparative semantic-spacing pattern persists under both missing-data treatments. In the observed-joint-only analysis, the proposed method obtains *CVsd* = 0.5072 ± 0.1387 and *MSD* = 1.2326 ± 0.1416; the most favorable baseline values are 1.6189 ± 0.0550 and 0.3087 ± 0.0322, respectively, both from uniform sampling. This corresponds to a 68.7% reduction in *CVsd* and a 299.3% increase in *MSD* relative to the strongest baseline on each metric. In the confidence-weighted analysis, the proposed method obtains *CVsd* = 0.5011 ± 0.1370 and *MSD* = 1.2221 ± 0.1373, compared with the best baseline values of 1.5688 ± 0.0485 and 0.3144 ± 0.0309, again from uniform sampling; the corresponding changes are 68.1% and 288.7%. *Etr* remains less favorable than the best reconstruction baselines (0.0902 versus 0.0767 in the observed-joint-only analysis and 0.0911 versus 0.0797 in the confidence-weighted analysis), preserving the reconstruction-versus-pose-spacing trade-off. Linear interpolation therefore affects the absolute metric values, but it does not account for the baseline-relative *CVsd* and *MSD* separation.

## 4. Discussion

The results demonstrate that pose-state organization is a meaningful and measurable keyframe objective for fixed-view assembly video. At 271 frames per analyzed file, the proposed method obtains *CVsd* = 0.3340 ± 0.1117, *KR* = 0.6031 ± 0.0009, and *MSD* = 0.7781 ± 0.1003. Relative to MViT-V2-S feature clustering, this corresponds to a 68.7% reduction in semantic-distance variation and a 162.8% increase in mean adjacent skeleton distance. In practical terms, the retained sequence changes pose state more evenly and avoids repeatedly spending the limited budget on closely related operator configurations.

The event results clarify rather than negate this contribution. Uniform, motion-adaptive, pixel-clustering, and MViT baselines retain more annotated process transitions because regular temporal allocation and appearance-motion cues are better aligned with phase-boundary coverage. The proposed method instead prioritizes diversity and regularity of pose states. These complementary outcomes show that keyframe quality is objective-dependent: pose-oriented browsing or annotation preparation may value differentiated configurations, whereas event auditing should use an event-aware selector or combine pose diversity with boundary cues.

The ablation supplies the central design insight: the skeleton representation, not merely a changing threshold, produces most of the semantic-spacing effect. Adaptive thresholding remains useful as an interpretable density-control device, although its incremental changes are small and budget-dependent. This distinction strengthens the method’s contribution by identifying which component should be retained when the framework is transferred or simplified.

The robustness and missing-data analyses define actionable operating guidance. Neck and hip anchors lead to closely overlapping outputs; moderate synthetic dropout and jitter preserve most selected locations; and the decision stage is lightweight after pose extraction. The matched-baseline observed-joint analyses show that interpolation contributes to lower *Etr* and *CVsd*, but excluding or downweighting missing joints does not remove the proposed method’s relative *CVsd* and *MSD* separation. At the same time, the less favorable *Etr* and the low jointly observed coverage require the result to be interpreted as an objective-specific pose-spacing advantage rather than unconditional superiority. Deployments should therefore monitor pose confidence and long missing gaps.

### Limitations and Open Questions

The evidence is limited to three temporally non-overlapping segments with different operation content from one longer recording with the same fixed monocular camera, workstation, operator, task setting, and acquisition environment. The segments are treated as descriptive within-recording test cases rather than independent recording-level replications. Accordingly, variation across segments is interpreted descriptively, while the temporally dependent event annotations are not treated as independent population-level observations. The design cannot establish cross-operator, cross-camera, cross-task, cross-lighting, or cross-site generalization. Experiments using additional viewpoints and independently recorded industrial videos were not feasible within the resources available for the present study because additional cameras and viewpoints would substantially increase the combined costs of video acquisition, storage, backup, transfer, computation, preprocessing, and manual annotation. These resource and cost constraints explain the bounded case-study design but do not substitute for future external validation.

The independent second human annotation and documented adjudication improve the transparency of the event reference. Pooled file-level temporal-boundary F1 values across the three distinct segments are 84.51% at ±0.5 s and 90.14% at ±1.0 s. Phase-pair agreement is lower, indicating sensitivity to annotation granularity. The missing-data sensitivity analysis further shows that interpolation lowers *Etr* and *CVsd*; semantic regularity is therefore partly preprocessing-dependent. The proposed method also provides lower process-transition coverage than several temporal, appearance-based, and pretrained baselines. Its favorable *CVsd*, *KR*, and *MSD* values are calculated in the same normalized skeleton space used for selection and should be interpreted as representation-aligned diagnostics rather than independent evidence of practical superiority. The ablation further shows that adaptive thresholding provides only small, budget-dependent changes. Robustness to real camera motion and usefulness in downstream industrial tasks have not been established. Remaining open questions include downstream validation, independent production-line evaluation, stronger missing-data handling, real camera-motion testing, and replacement of OpenPose with a lightweight pose estimator. The measured 16.19 FPS on an RTX 4090D remains below the 24-FPS source rate, so the implementation is positioned only as offline preprocessing.

## 5. Conclusions

This study designs and evaluates a deterministic, training-free framework for converting continuous fixed-view assembly video into a compact sequence of differentiated operator-pose states. Its value lies in a combination that is often important in industrial preprocessing but not simultaneously provided by learned summarizers: no task-specific training requirement, an explicit and reproducible decision rule, direct output-budget control, and a pose-centered representation that reduces the influence of irrelevant appearance changes. Under matched budgets, the method produces markedly more regular skeleton-semantic increments and greater adjacent pose separation than the evaluated baselines. The ablation identifies skeleton representation as the decisive component, while event evaluation shows that pose diversity and phase-boundary coverage are complementary objectives rather than interchangeable definitions of quality. The two-annotator audit, deterministic capacity rule, degradation tests, and matched-baseline observed-joint sensitivity make the demonstrated behavior reproducible and show that the comparative pose-spacing pattern is not attributable solely to interpolation. The present evidence supports use as an interpretable pose-oriented preprocessing strategy in the evaluated fixed-view setting. Future work will test whether combining pose diversity with event cues improves downstream assembly understanding and will evaluate transfer across independently recorded operators, cameras, tasks, and sites.

## Figures and Tables

**Table 1:** Main notation used in the proposed skeleton-semantic keyframe extraction method.

Symbol	Definition
$I_{t}$	The t−th frame of the input industrial assembly video
N	Total number of video frames
$p_{\left\{t,j\right\}}$	Two-dimensional coordinate of the j-th BODY-25 joint in frame t
$c_{\left\{t,j\right\}}$	Confidence score of the j-th joint in frame t
$c_{min}$	Confidence threshold for valid skeleton keypoint detection
$J_{f}$	Joint index set used for skeleton feature construction
$J_{m}$	Joint index set used for motion activity estimation
$f_{t}$	Skeleton feature vector of frame t
$b_{t}$	Anchor point used for relative motion calculation
$m_{t}$	Relative motion magnitude at frame t
${\bar{m}}_{t}$	Smoothed motion magnitude after sliding-window filtering
$a_{t}$	Normalized motion activity coefficient
$T_{t}$	Adaptive threshold at frame t
$T_{min}$	Lower bound of the adaptive threshold
$T_{max}$	Upper bound of the adaptive threshold
$d_{t}$	Feature distance between the current frame and the reference keyframe
K	Output keyframe index set
W	Sliding-window size
$G_{max}$	Maximum allowed frame gap between two adjacent keyframes

**Table 2:** Pseudocode of the proposed skeleton-semantic adaptive keyframe extraction method.

Line	Operation
Input	Video frames; confidence threshold $c_{min};$ selected joint set $J_{f};$ wrist-joint set $J_{m};$ sliding-window size W; maximum frame gap $G_{max};$ target keyframe count.
Output	$\mathrm{Keyframe}\;\mathrm{index}\;\mathrm{set}\;K_{f}.$
1	Extract BODY-25 keypoints and confidence scores from every frame using OpenPose.
2	Form the confidence-validity mask, normalize the complete BODY-25 coordinate sequence, and temporally interpolate low-confidence normalized coordinates.
3	Select the upper-body joints $J_{f}$ and concatenate their normalized coordinates to construct the frame feature $f_{t}.$
4	Use the neck joint as the default anchor; use the hip center only in the anchor-comparison experiment.
5	Calculate bilateral-wrist motion relative to the anchor and smooth the motion magnitude with a W-frame mean filter.
6	Normalize the smoothed motion magnitude to obtain the activity coefficien $a_{t}.$
7	Determine $T_{min}$ by 30-iteration binary search over [0.01, 3.0], set $T_{max}$$=\;2.5\;T_{min},$ $\mathrm{and}\;\mathrm{generate}\;T_{t}$ using the inverse activity-threshold mapping.
8	$\mathrm{Initialize}\;K_{f}$ with the first frame and use its feature as the reference feature.
9	For each subsequent frame, calculate $d_{t}.$ $\mathrm{If}\;d_{t}$ $\geq \;T_{t}$ or the frame gap exceeds $G_{max},$ add the frame to $K_{f}$ and update the reference feature.
10	When an exact budget B is required, retain the binary-search output with the smallest |N − B| (ties: earlier search iteration). If N < B, add unselected frames in ascending order of $\left|d_{t}-T_{t}\right|$ (ties: lower frame index). If N > B, rank non-endpoint selected frames by the sum of distances to their adjacent selected frames; remove in ascending order only when the resulting gap is <=*G_max_* (ties: lower frame index). Do not rerun sequential reference updates; stop at N = B and return K.

**Table 3:** Parameter settings used in this study.

Parameter	Value	Description
$c_{min}$	0.2	Confidence threshold for valid skeleton keypoints
W	15	Sliding-window size for temporal smoothing
ε	10^−5^	Small threshold for suppressing numerical fluctuations
$G_{max}$	50	Maximum allowed frame gap between adjacent keyframes
$J_{f}$	{1, 2, 3, 4, 5, 6, 7, 8}	Joint set for skeleton feature construction
$J_{m}$	{4, 7}	Wrist-joint set for motion activity estimation
Anchor	Neck (default); hip center (comparison)	Anchor used for relative wrist-motion calculation
$T_{max}$ $/T_{min}$	2.5	Adaptive-threshold bound ratio
$T_{min}$ search interval	[0.01, 3.0]	Binary-search interval for capacity tuning
Search iterations	30	Fixed number of binary-search iterations

**Table 4:** Dataset statistics of the industrial assembly videos used in the experiments.

Dataset	Video File	Frames	Duration	FPS	Resolution	Motion Intensity	Main Operation Characteristics
Group-1	video951.mp4	2882	00:02:00	24.00	640 × 368	Low-to-medium	Repeated material handling, label/material preparation, positioning, and short stable assembly intervals
Group-2	video2.mp4	2938	00:02:02	24.00	640 × 368	Medium	Fine assembly and component positioning with more frequent wrist movement and operation transitions
Group-3	video3.mp4	3207	00:02:13	24.00	640 × 368	Medium-to-high	Longer sequence with material handling, side reaching, workpiece transfer, and more missing-keypoint cases

**Table 5:** Experimental environment configuration.

Environment	Parameter	Specification
Keyframe extraction and post-processing	CPU	Intel Core i7-11700K @ 3.60 GHz
Keyframe extraction and post-processing	Operating system	Microsoft Windows 10 (64-bit)
Keyframe extraction and post-processing	Pose estimator	OpenPose v1.7.0, BODY-25
End-to-end runtime benchmark	Cloud platform	AutoDL Linux container
End-to-end runtime benchmark	CPU	Intel Xeon Platinum 8474C
End-to-end runtime benchmark	GPU	NVIDIA GeForce RTX 4090D, 24 GB
End-to-end runtime benchmark	Driver/CUDA	Driver 580.105.08/CUDA 13.0
End-to-end runtime benchmark	Python	Python 3.8.10/NumPy 1.23.3
End-to-end runtime benchmark	OpenPose setting	BODY-25; net resolution −1 × 368; one person
Modern transformer baseline	Transformer software	PyTorch 2.9.0/torchvision 0.24.0; CPU inference
Modern transformer baseline	Transformer model	MViT-V2-S; Kinetics-400 pretrained; no fine-tuning
Modern transformer baseline	Transformer input	96 clips/segment; 16 frames/clip; temporal stride 3; 224 × 224 crop

**Table 6:** Keyframe extraction performance on the three industrial assembly video segments.

Dataset	Original Frames	Keyframes	CR	Etr	TC	CVsd	KR	MSD
Group-1	2881	271	90.59%	0.1006	89.74%	0.2289	0.6023	0.8877
Group-2	2938	270	90.81%	0.0604	93.93%	0.4467	0.6044	0.6934
Group-3	3207	271	91.55%	0.0748	92.49%	0.3218	0.6029	0.7556

**Table 7:** Matched-budget comparison at 271 keyframes per segment (mean ± standard deviation across three video segments).

Algorithm	Feature Space	Frames/ Segment	Etr	TC (%)	CVsd	KR	MSD
Proposed method	Skeleton	271	0.0786 ± 0.0204	92.05 ± 2.13	0.3340 ± 0.1117	0.6031 ± 0.0009	0.7781 ± 0.1003
Uniform sampling	Temporal index	271	0.0633 ± 0.0081	93.61 ± 0.87	1.0140 ± 0.0924	0.6701 ± 0.0164	0.3100 ± 0.0125
Pose-space clustering	Pose	271	0.0678 ± 0.0068	93.15 ± 0.75	1.1498 ± 0.0353	0.6875 ± 0.0076	0.2829 ± 0.0160
Pixel-space clustering	Decoded pixels	271	0.0686 ± 0.0065	93.07 ± 0.72	1.0595 ± 0.0633	0.6839 ± 0.0122	0.2867 ± 0.0150
Fixed-threshold (pixel MSE)	Decoded pixels	271	0.1015 ± 0.0169	89.76 ± 1.65	1.5184 ± 0.1122	0.7497 ± 0.0138	0.1931 ± 0.0054
ORB + Flow	Decoded pixels	271	0.1359 ± 0.0052	86.29 ± 0.44	2.0589 ± 0.1720	0.7990 ± 0.0171	0.1087 ± 0.0177
Motion-adaptive	Decoded pixels	271	0.0767 ± 0.0032	92.26 ± 0.36	1.1533 ± 0.0034	0.7020 ± 0.0057	0.2454 ± 0.0123
MViT-V2-S feature clustering	Pretrained video transformer	271	0.0645 ± 0.0068	93.48 ± 0.74	1.0681 ± 0.0754	0.6822 ± 0.0160	0.2961 ± 0.0305

**Table 8:** Matched-budget comparison against 34 manually annotated assembly-process transitions across the three video segments.

Budget per Segment	Method	Total Keyframes	Events	ER at ±0.5 s (%)	Mean Nearest-Keyframe Error (s)
60	Proposed method	180	34	41.18	0.602
60	Uniform sampling	180	34	52.94	0.499
60	Pose-space clustering	180	34	41.18	0.825
60	Pixel-space clustering	180	34	44.12	0.661
60	Fixed-threshold (pixel MSE)	180	34	35.29	1.534
60	ORB + Flow	180	34	32.35	1.983
60	Motion-adaptive	180	34	70.59	0.413
60	MViT-V2-S feature clustering	180	34	61.76	0.518
120	Proposed method	360	34	58.82	0.482
120	Uniform sampling	360	34	97.06	0.243
120	Pose-space clustering	360	34	67.65	0.478
120	Pixel-space clustering	360	34	91.18	0.254
120	Fixed-threshold (pixel MSE)	360	34	61.76	0.618
120	ORB + Flow	360	34	35.29	1.741
120	Motion-adaptive	360	34	97.06	0.168
120	MViT-V2-S feature clustering	360	34	94.12	0.219
271	Proposed method	813	34	79.41	0.259
271	Uniform sampling	813	34	100.00	0.107
271	Pose-space clustering	813	34	97.06	0.148
271	Pixel-space clustering	813	34	100.00	0.102
271	Fixed-threshold (pixel MSE)	813	34	94.12	0.165
271	ORB + Flow	813	34	38.24	1.597
271	Motion-adaptive	813	34	97.06	0.089
271	MViT-V2-S feature clustering	813	34	100.00	0.120

**Table 9:** Matched-budget ablation results across the three industrial assembly video segments (mean ± standard deviation).

Budget	Variant	Etr	TC (%)	CVsd	MSD
60	A: Pixel + Static	0.0951 ± 0.0024	90.40 ± 0.27	0.6477 ± 0.1129	0.5311 ± 0.0823
60	B: Skeleton + Static	0.0963 ± 0.0096	90.28 ± 1.06	0.6300 ± 0.0932	0.6004 ± 0.0267
60	C: Pixel + Adaptive	0.0946 ± 0.0051	90.44 ± 0.60	0.6289 ± 0.0957	0.5601 ± 0.0547
60	D: Skeleton + Adaptive	0.0990 ± 0.0076	90.00 ± 0.85	0.5883 ± 0.0669	0.6105 ± 0.0422
120	A: Pixel + Static	0.0852 ± 0.0043	91.40 ± 0.51	0.8315 ± 0.0312	0.4179 ± 0.0096
120	B: Skeleton + Static	0.0971 ± 0.0058	90.20 ± 0.65	0.4089 ± 0.0094	0.8415 ± 0.0253
120	C: Pixel + Adaptive	0.0893 ± 0.0050	90.98 ± 0.58	0.7779 ± 0.0275	0.4289 ± 0.0364
120	D: Skeleton + Adaptive	0.0981 ± 0.0105	90.09 ± 1.14	0.4320 ± 0.0230	0.8123 ± 0.0042
271	A: Pixel + Static	0.0739 ± 0.0059	92.54 ± 0.66	1.0907 ± 0.0562	0.2747 ± 0.0168
271	B: Skeleton + Static	0.0794 ± 0.0201	91.97 ± 2.11	0.3439 ± 0.1093	0.7692 ± 0.1025
271	C: Pixel + Adaptive	0.0720 ± 0.0069	92.73 ± 0.75	1.0815 ± 0.0399	0.2935 ± 0.0274
271	D: Skeleton + Adaptive	0.0784 ± 0.0197	92.08 ± 2.06	0.3301 ± 0.1147	0.7830 ± 0.1057

**Table 10:** Parameter sensitivity results across the three industrial assembly video segments (mean ± standard deviation).

Parameter	Value	Keyframes	Etr	CVsd	MSD	ER ± 0.5 s (%)
W	5	265.3 ± 2.9	0.0797 ± 0.0190	0.3256 ± 0.1076	0.7882 ± 0.1001	77.31 ± 10.42
W	15	270.7 ± 0.6	0.0786 ± 0.0204	0.3324 ± 0.1093	0.7789 ± 0.0992	77.31 ± 10.42
W	25	271.7 ± 3.2	0.0782 ± 0.0202	0.3366 ± 0.1124	0.7769 ± 0.1070	77.31 ± 10.42
W	35	272.3 ± 4.0	0.0784 ± 0.0194	0.3394 ± 0.1151	0.7735 ± 0.1053	77.31 ± 10.42
$c_{min}$	0.1	273.0 ± 16.7	0.0874 ± 0.0151	0.3371 ± 0.0987	0.8255 ± 0.0796	84.72 ± 6.05
$c_{min}$	0.2	270.7 ± 0.6	0.0786 ± 0.0204	0.3324 ± 0.1093	0.7789 ± 0.0992	77.31 ± 10.42
$c_{min}$	0.3	256.3 ± 4.7	0.0736 ± 0.0199	0.3226 ± 0.0808	0.7282 ± 0.0888	77.31 ± 10.42
$c_{min}$	0.4	232.0 ± 16.5	0.0716 ± 0.0203	0.2921 ± 0.0955	0.6703 ± 0.1007	73.61 ± 16.37
epsilon	0	270.7 ± 0.6	0.0786 ± 0.0204	0.3324 ± 0.1093	0.7789 ± 0.0992	77.31 ± 10.42
epsilon	1 × 10^−6^	270.7 ± 0.6	0.0786 ± 0.0204	0.3324 ± 0.1093	0.7789 ± 0.0992	77.31 ± 10.42
epsilon	1 × 10^−5^	270.7 ± 0.6	0.0786 ± 0.0204	0.3324 ± 0.1093	0.7789 ± 0.0992	77.31 ± 10.42
epsilon	1 × 10^−4^	270.7 ± 0.6	0.0786 ± 0.0204	0.3324 ± 0.1093	0.7789 ± 0.0992	77.31 ± 10.42
$G_{max}$	30	293.0 ± 7.9	0.0703 ± 0.0150	0.4075 ± 0.0910	0.7333 ± 0.0930	86.81 ± 8.19
$G_{max}$	50	270.7 ± 0.6	0.0786 ± 0.0204	0.3324 ± 0.1093	0.7789 ± 0.0992	77.31 ± 10.42
$G_{max}$	75	254.0 ± 10.1	0.0908 ± 0.0252	0.3003 ± 0.1197	0.8006 ± 0.1055	68.98 ± 7.90
$G_{max}$	100	254.3 ± 3.5	0.0955 ± 0.0252	0.2806 ± 0.1226	0.8111 ± 0.1046	61.57 ± 5.61
$T_{min}$ multiplier	0.75	273.0 ± 2.6	0.0777 ± 0.0187	0.3356 ± 0.1084	0.7759 ± 0.0974	77.31 ± 10.42
$T_{min}$ multiplier	1	270.7 ± 0.6	0.0786 ± 0.0204	0.3324 ± 0.1093	0.7789 ± 0.0992	77.31 ± 10.42
$T_{min}$ multiplier	1.25	269.3 ± 1.5	0.0785 ± 0.0206	0.3293 ± 0.1094	0.7809 ± 0.1010	77.31 ± 10.42
$T_{max}$ multiplier	0.75	306.7 ± 4.0	0.0744 ± 0.0193	0.4000 ± 0.1236	0.7134 ± 0.1074	86.34 ± 11.95
$T_{max}$ multiplier	1	270.7 ± 0.6	0.0786 ± 0.0204	0.3324 ± 0.1093	0.7789 ± 0.0992	77.31 ± 10.42
$T_{max}$ multiplier	1.25	241.0 ± 5.6	0.0867 ± 0.0166	0.3066 ± 0.0802	0.8252 ± 0.0870	73.15 ± 5.78

**Table 11:** Comparison of neck-anchor and hip-center-anchor configurations under the same method settings.

Dataset	Anchor	Frames	Keyframes	CR (%)	Etr	TC (%)	CVsd	MSD
Group-1	Neck	2881	271	90.59	0.1006	89.74	0.2289	0.8877
Group-1	Hip center	2881	270	90.63	0.1013	89.68	0.2259	0.8919
Group-2	Neck	2938	270	90.81	0.0604	93.93	0.4467	0.6934
Group-2	Hip center	2938	271	90.78	0.0651	93.47	0.4513	0.6921
Group-3	Neck	3207	271	91.55	0.0748	92.49	0.3218	0.7556
Group-3	Hip center	3207	272	91.52	0.0732	92.65	0.3246	0.7495

**Table 12:** Event-level comparison of the default neck-anchor method and hip-center variant against the adjudicated 34-transition consensus reference.

Dataset	Configuration	Keyframes	Events	ER at ±0.5 s (%)	Matched-Event MTE at ±0.5 s (s)	ER at ±1.0 s (%)	Matched-Event MTE at ±1.0 s (s)
Group-1	Neck anchor (default)	271	9	66.67	0.042	100.00	0.269
Group-1	Hip-center anchor	270	9	66.67	0.042	100.00	0.264
Group-2	Neck anchor (default)	270	9	77.78	0.143	100.00	0.278
Group-2	Hip-center anchor	271	9	77.78	0.149	100.00	0.282
Group-3	Neck anchor (default)	271	16	87.50	0.167	93.75	0.239
Group-3	Hip-center anchor	272	16	87.50	0.173	93.75	0.244
Overall	Neck anchor (default)	812	34	79.41	0.133	97.06	0.258
Overall	Hip-center anchor	813	34	79.41	0.137	97.06	0.260

**Table 13:** Sensitivity of normalized motion-activity estimation and keyframe selection to neck and hip-center anchors.

Dataset	Activity r	Activity MAE	Activity RMSE	P95 Absolute Discrepancy	Top-10% Activity Overlap (%)	Keyframe Overlap Within ±3 Frames (%)
Group-1	0.743	0.0736	0.1233	0.2846	44.14	95.20
Group-2	0.748	0.0756	0.1359	0.3252	46.63	92.99
Group-3	0.660	0.0461	0.1140	0.2465	39.78	97.79

**Table 14:** Controlled skeleton-degradation and global camera-motion results across the three video segments and repeated random seeds (mean ± standard deviation).

Condition	Level	Runs	Keyframes	Etr	ER ± 0.5 s (%)	Baseline Overlap (%)
Baseline	None	3	270.7 ± 0.6	0.0786 ± 0.0204	77.31 ± 10.42	100.00 ± 0.00
Additional wrist dropout	10%	30	263.9 ± 5.1	0.0791 ± 0.0167	77.11 ± 8.48	97.54 ± 1.61
Additional wrist dropout	20%	30	257.8 ± 6.6	0.0800 ± 0.0163	77.43 ± 7.62	95.10 ± 2.47
Additional wrist dropout	40%	30	248.4 ± 10.3	0.0819 ± 0.0157	77.18 ± 7.49	91.07 ± 2.91
Upper-body occlusion	10%	30	247.9 ± 7.2	0.0802 ± 0.0159	76.90 ± 9.09	90.54 ± 2.64
Upper-body occlusion	20%	30	226.2 ± 12.5	0.0825 ± 0.0143	74.00 ± 10.06	80.63 ± 5.26
Upper-body occlusion	30%	30	206.1 ± 13.8	0.0837 ± 0.0128	71.39 ± 9.76	72.98 ± 4.82
Coordinate jitter	sigma = 0.005	30	272.2 ± 2.6	0.0789 ± 0.0165	77.31 ± 8.66	99.05 ± 0.94
Coordinate jitter	sigma = 0.01	30	273.4 ± 2.9	0.0786 ± 0.0164	77.69 ± 8.42	97.95 ± 1.26
Coordinate jitter	sigma = 0.02	30	279.0 ± 5.5	0.0783 ± 0.0172	80.65 ± 10.38	96.09 ± 1.75
Global camera vibration	Mild (3.2 px, 0.25 deg)	30	271.3 ± 1.6	0.0787 ± 0.0165	77.31 ± 8.66	99.08 ± 0.79
Global camera vibration	Moderate (6.4 px, 0.50 deg)	30	271.0 ± 2.5	0.0788 ± 0.0162	77.31 ± 8.66	98.28 ± 1.14
Global camera vibration	Strong (12.8 px, 1.00 deg)	30	271.0 ± 2.7	0.0790 ± 0.0160	77.31 ± 8.66	97.06 ± 1.21

**Table 15:** Internal illumination diagnostics from independently rerun OpenPose sequences across the three segments (mean ± standard deviation).

Condition	Detection Rate (%)	Feature-Joint Missing Rate (%)	Wrist-Joint Missing Rate (%)	Keyframe Overlap Within ±3 Frames (%)
Original-lighting rerun	98.93 ± 0.25	42.43 ± 2.81	27.21 ± 2.34	100.00 ± 0.00
Dark ×0.5	97.93 ± 0.86	50.49 ± 1.36	33.56 ± 2.83	57.07 ± 4.82
Overexposed ×1.5	91.36 ± 4.22	63.09 ± 3.30	42.58 ± 8.32	42.80 ± 4.53
Low contrast ×0.6	98.98 ± 0.30	42.92 ± 1.53	29.18 ± 2.26	60.64 ± 4.40

**Table 16:** Computational cost and missing-keypoint statistics of the skeleton-based decision stage.

Dataset	Anchor	Processing Time (s)	FPS (Excluding CSV Load)	Peak Memory (MB)	Feature-Joint Missing Rate	Wrist-Joint Missing Rate
Group-1	Neck	3.525	817.4	7.57	33.75%	26.07%
Group-1	Hip center	4.049	711.6	7.67	33.75%	26.07%
Group-2	Neck	3.637	807.7	7.72	39.26%	27.93%
Group-2	Hip center	4.206	698.6	7.81	39.26%	27.93%
Group-3	Neck	3.907	820.8	8.41	50.00%	36.22%
Group-3	Hip center	4.436	723.0	8.51	50.00%	36.22%

**Table 17:** OpenPose GPU and end-to-end runtime benchmark on the AutoDL RTX 4090D environment (mean ± standard deviation over three runs).

Dataset	Frames/Run	Detection Rate (%)	OpenPose FPS	End-to-End FPS	Latency (ms/Frame)	Peak GPU Memory Increase (MB)
Group-1	300	99.00 ± 0.00	16.54 ± 1.35	16.35 ± 1.26	61.41 ± 4.55	10,489 ± 0
Group-2	300	100.00 ± 0.00	17.17 ± 1.64	16.99 ± 1.61	59.19 ± 5.38	10,489 ± 0
Group-3	300	99.67 ± 0.00	15.36 ± 0.68	15.22 ± 0.67	65.77 ± 2.85	10,489 ± 0
Overall	300	99.56 ± 0.44	16.36 ± 1.37	16.19 ± 1.33	62.12 ± 4.78	10,489 ± 0

**Table 18:** Pooled missing-gap lengths across the eight BODY-25 feature joints at confidence < 0.2.

File	Gaps	Median (Frames/s)	IQR (Frames)	P95 (Frames/s)	Maximum (Frames/s)	>0.5 s (%)	>1.0 s (%)
Group-1	2099	2/0.083	2	11/0.458	155/6.458	4.48	1.67
Group-2	1446	2/0.083	4	25/1.042	150/6.250	10.44	5.12
Group-3	1584	2/0.083	5	33/1.375	366/15.250	15.09	7.70

**Table 19:** Matched-baseline missing-data sensitivity at 271 keyframes per analyzed file (mean ± sample standard deviation across the three files).

Method	Missing-Data Evaluation	Etr	CVsd	MSD
Proposed method	Observed-joint-only	0.0902 ± 0.0197	0.5072 ± 0.1387	1.2326 ± 0.1416
Proposed method	Confidence-weighted	0.0911 ± 0.0195	0.5011 ± 0.1370	1.2221 ± 0.1373
Uniform sampling	Observed-joint-only	0.0767 ± 0.0070	1.6189 ± 0.0550	0.3087 ± 0.0322
Uniform sampling	Confidence-weighted	0.0797 ± 0.0084	1.5688 ± 0.0485	0.3144 ± 0.0309
Pose-space clustering	Observed-joint-only	0.0787 ± 0.0047	1.7235 ± 0.0234	0.2777 ± 0.0155
Pose-space clustering	Confidence-weighted	0.0819 ± 0.0057	1.6781 ± 0.0287	0.2822 ± 0.0161
Pixel-space clustering	Observed-joint-only	0.0821 ± 0.0055	1.6722 ± 0.0911	0.2600 ± 0.0467
Pixel-space clustering	Confidence-weighted	0.0850 ± 0.0066	1.6141 ± 0.0865	0.2667 ± 0.0460
Fixed-threshold (pixel MSE)	Observed-joint-only	0.1067 ± 0.0176	2.1307 ± 0.0335	0.2083 ± 0.0130
Fixed-threshold (pixel MSE)	Confidence-weighted	0.1118 ± 0.0174	2.1036 ± 0.0296	0.2128 ± 0.0145
ORB + Flow	Observed-joint-only	0.1401 ± 0.0070	2.8162 ± 0.1927	0.1126 ± 0.0428
ORB + Flow	Confidence-weighted	0.1444 ± 0.0101	2.8000 ± 0.1954	0.1147 ± 0.0425
Motion-adaptive	Observed-joint-only	0.0874 ± 0.0031	1.8488 ± 0.0231	0.2389 ± 0.0202
Motion-adaptive	Confidence-weighted	0.0911 ± 0.0036	1.8043 ± 0.0154	0.2447 ± 0.0206
MViT-V2-S feature clustering	Observed-joint-only	0.0789 ± 0.0074	1.7212 ± 0.0641	0.2929 ± 0.0309
MViT-V2-S feature clustering	Confidence-weighted	0.0821 ± 0.0080	1.6753 ± 0.0543	0.2986 ± 0.0293

## Data Availability

The source code is not publicly available because it forms part of an ongoing research project and is subject to intellectual-property and industrial-confidentiality restrictions. To support reproducibility, the manuscript provides the complete algorithmic procedure, pseudocode, parameter settings, baseline definitions, and evaluation protocol. The implementation code may be considered for access upon reasonable request to the corresponding author, subject to authorization by the relevant rights holders. Derived skeleton data and keyframe indices may likewise be requested subject to confidentiality and participant-consent restrictions. The original industrial video is not publicly released because of workplace confidentiality and privacy constraints.
